# Rapid risk assessment tool (RRAT) to prioritize emerging and re-emerging livestock diseases for risk management

**DOI:** 10.3389/fvets.2022.963758

**Published:** 2022-09-07

**Authors:** Clazien J. de Vos, Ronald Petie, Ed G. M. van Klink, Manon Swanenburg

**Affiliations:** Wageningen Bioveterinary Research, Wageningen University & Research, Lelystad, Netherlands

**Keywords:** incursion risk, animal trade, animal products, travelers, livestock diseases, risk ranking, Netherlands

## Abstract

Increasing globalization and international trade contribute to rapid expansion of animal and human diseases. Hence, preparedness is warranted to prevent outbreaks of emerging and re-emerging diseases or detect outbreaks in an early stage. We developed a rapid risk assessment tool (RRAT) to inform risk managers on the incursion risk of multiple livestock diseases, about the main sources for incursion and the change of risk over time. RRAT was built as a relational database to link data on disease outbreaks worldwide, on introduction routes and on disease-specific parameters. The tool was parameterized to assess the incursion risk of 10 livestock diseases for the Netherlands by three introduction routes: legal trade in live animals, legal trade of animal products, and animal products illegally carried by air travelers. RRAT calculates a semi-quantitative risk score for the incursion risk of each disease, the results of which allow for prioritization. Results based on the years 2016-2018 indicated that the legal introduction routes had the highest incursion risk for bovine tuberculosis, whereas the illegal route posed the highest risk for classical swine fever. The overall incursion risk *via* the illegal route was lower than *via* the legal routes. The incursion risk of African swine fever increased over the period considered, whereas the risk of equine infectious anemia decreased. The variation in the incursion risk over time illustrates the need to update the risk estimates on a regular basis. RRAT has been designed such that the risk assessment can be automatically updated when new data becomes available. For diseases with high-risk scores, model results can be analyzed in more detail to see which countries and trade flows contribute most to the risk, the results of which can be used to design risk-based surveillance. RRAT thus provides a multitude of information to evaluate the incursion risk of livestock diseases at different levels of detail. To give risk managers access to all results of RRAT, an online visualization tool was built.

## Introduction

Increasing globalization and international trade contribute to rapid expansion of animal and human diseases. Introduction of animal diseases into naive livestock populations can result in large-scale epidemics with serious economic and socio-ethical impact. Illustrative examples include the introduction of foot and mouth disease (FMD) in the United Kingdom in 2001 (1) and subsequent spread to the Netherlands (2), the introduction of bluetongue (BT) in the Netherlands in 2006 with subsequent spread to neighboring countries (3, 4), and the introduction of African swine fever (ASF) into Georgia in 2007 (5). ASF subsequently spread into Europe and Asia (6), and in 2021 the disease was also introduced into the Americas (7). Recent incursions of diseases that had not been reported before in Europe, such as lumpy skin disease (LSD) in South-Eastern Europe in 2014-2017 and peste des petits ruminants (PPR) in Bulgaria in 2018 highlight the continuous threat of emerging and re-emerging disease outbreaks (7–9). Preparedness is thus warranted to prevent outbreaks of livestock diseases in new territories or to detect outbreaks in an early stage. Risk assessment is a useful tool to inform risk managers on the incursion risk of livestock diseases that can not only provide information on the magnitude of the risk, but also on the main sources of risk and the change of risk over time.

Most introduction risk assessments performed over the last decades focused on a single disease and a single introduction route [e.g., (10–15)] and were initiated to address specific risk questions. Those risk questions often arise in response to new disease events to evaluate the increased incursion risk from such an event. In recent years, several generic risk assessment tools were developed that accommodate multiple diseases and/or introduction routes (16). An important asset of these generic risk assessment tools is the ability to prioritize diseases or risk regions for their incursion risk, enabling the targeted use of limited resources for prevention and surveillance. The repeated use of these tools to inform risk managers is, however, limited, either because the tools do not have an underlying database and need to be populated with data before each use, or because expert opinion is needed to update results. One of the earliest prioritization tools was developed by Roberts et al. (17) for the United Kingdom. This tool integrates expert opinion with data on disease outbreaks and trade into a semi-quantitative risk score for each disease. Updates are performed manually, and expert opinion is key to keep the tool up and running. An automatic update of data and calculations when new data becomes available would facilitate repeated use of generic risk assessment tools.

The incursion risk of a livestock disease is largely determined by the distribution of the pathogen in the world and the connections of a disease-free territory with these regions. These connections are the so-called introduction routes and can either be trade in livestock or their products, trade in exotic animals, migrating wildlife, movements of people if the disease is zoonotic, or introduction of vectors if the disease is vector-borne. Data on the worldwide distribution of animal diseases and on the volume of introduction routes is largely available from global databases such as WAHIS (World Animal Health Information System) (7), EMPRES-i (Global Animal Disease Information System) (18), Comtrade (19) and Comext (20). Integration of this data is mostly done by disease experts leaving it a labor-intensive and subjective exercise to evaluate the incursion risk. The increased accessibility and interoperability of most of these global databases has opened the door to a more automated risk assessment approach. To fully exploit the available data, we developed a rapid risk assessment tool (RRAT) that combines the data from global databases into an automated estimate of the incursion risk for multiple livestock diseases. The main objective of this tool is to support risk managers in prioritizing diseases for risk management. Furthermore, RRAT can indicate high risk trade flows and source countries, the results of which can be used for risk-based surveillance. In this paper, RRAT is described and results for the incursion risk for the Netherlands are presented and discussed.

## Materials and methods

RRAT has been built as a relational database in R (21) and SQLite (22) with the main tables in the tool describing: the worldwide occurrence of animal diseases; the volumes of the introduction routes; and disease-specific parameters to assess the risk of each introduction route. RRAT is a semi-quantitative risk assessment tool that provides the user with a risk score for the probability that a specific disease enters a new region or country (“target area”) and will result in a first infection of local livestock animals. Introduction routes considered in RRAT comprise the legal trade in live animals (“animal route”), the legal trade of animal products (“product route”), and animal products illegally carried by air travelers (“traveler route”). The introduction routes are all subdivided into multiple pathways to account for diversity in animal species and animal products. Up till now, RRAT has been parameterized for 10 diseases that are considered a potential threat to the Netherlands, viz. African horse sickness (AHS), ASF, Aujeszky's disease (Auj), BT, bovine tuberculosis (bTB), classical swine fever (CSF), equine infectious anemia (EIA), foot and mouth disease (FMD), LSD, and PPR (Table 1). Calculations have been performed for the years 2016, 2017 and 2018 with the Netherlands as target area.

**Table 1:** Overview of causing pathogens, reservoir livestock hosts and main transmission routes of ten diseases in RRAT.

**Disease**	**Pathogen^a^**	**Reservoir livestock host**	**Main transmission route**
African horse sickness	AHS virus (*Orbivirus, Reoviridae*)	Horses	Biological vector (*Culicoides* spp.)
African swine fever	ASF virus (*Asfivirus, Asfarviridae*)	Pigs	Direct and indirect contact, swill feeding, biological vector (*Ornithodorus* spp.)
Aujeszky's disease	Suid herpesvirus 1 (*Varicellovirus, Herpesviridae*)	Pigs	Direct and indirect contact, venereal transmission, swill feeding
Bluetongue	BT virus (*Orbivirus, Reoviridae*)	Bovines, sheep, goats	Biological vector (*Culicoides* spp.)
Bovine tuberculosis	*Mycobacteriumbovis* ^b^	Bovines, pigs, goats	Direct contact, respiratory transmission, ingestion of raw meat and milk
Classical swine fever	CSF virus (*Pestivirus, Flaviviridae*)	Pigs	Direct and indirect contact, venereal and congenital transmission, swill feeding
Equine infectious anemia	EIA virus (*Lentivirus, Retroviridae*)	Horses	Mechanical vectors (*Tabanidae* family, *Stomoxys calcitrans*)
Foot and mouth disease	FMD virus (*Aphthovirus, Picornaviridae*)	Bovines, pigs, sheep, goats	Direct and indirect contact, airborne transmission, swill feeding
Lumpy skin disease	LSD virus (*Capripoxvirus, Poxviridae*)	Bovines	Mechanical vectors (mosquitoes, biting flies, *Culicoides* spp., hard ticks), venereal and congenital transmission
Peste des petits ruminants	PPR virus (*Morbillivirus, Paramyxoviridae*)	Sheep, goats	Direct and indirect contact

^a^Genus and family of pathogen given between brackets.

^b^Some outbreaks of bovine tuberculosis are caused by *M. caprae*.

Calculations in RRAT are based on the Binomial process considering (1) the number of animals or products entering the target area, (2) the probability that an individual animal or product is infected, and (3) the probability that entry of an infected animal or product results in a first infection of local animals. An overview of the model parameters in RRAT is given in Table 2.

**Table 2:** Overview of model parameters in RRAT.

**Parameter**	**Description**	**Introduction route**	**Reference**
*N*_*CP*_	Number of pathway units of pathway *P* from source country *C*	Animal, Product, Traveler	Supplementary Tables S1.1; S1.2; S1.3
*P*_*entr*_*y*__*CPD*__	Probability of entry of disease *D* from source country *C* by pathway *P*	Animal, Product, Traveler	Eq. 3; Eq. 6
*P*_*est*_*PD*__	Probability that entry of disease *D* by pathway *P* results in a first local infection (establishment)	Animal, Product, Traveler	Eq. 5; Eq. 8
*Inc*_*CD*_	Incidence of disease *D* in source country *C*	Animal	(58)
*Inc*_*ab*_*s*__*D*__	Proxy value to estimate incidence of disease *D* for countries where disease is absent from domestic livestock (risk class 1, 2 or 3, Figure 3)	Animal	Supplementary Table S3.2
*Inc*_*un*_*k*__*D*__	Proxy value to estimate incidence of disease *D* for countries where presence of disease is unknown (risk class 5 or 6, Figure 3)	Animal	Supplementary Table S3.2
*P*_*infsu*_*s*__*P*__	Susceptibility-class dependent probability of infection of animal species *P*	Animal	Supplementary Table S3.1
*T*_*in*_*f*__*D*__	Average infectious period of disease *D* in reservoir hosts	Animal	Supplementary Table S3.2
*P*_*in*_*f*__*CPD*__	Probability of animal species *P* from country *C* being infected with disease *D*	Animal, Product	Eq. 4
*P*_*de*_*t*__*CPD*__	Probability of animal species *P* infected with disease *D* being detected before transport in country *C*	Animal	(31–38)
*P*_*contac*_*t*__*P*__	Probability that an imported infected animal of animal species *P* comes into contact with susceptible livestock	Animal	Supplementary Table S3.3
*P*_*tran*_*s*__*PD*__	Probability that an infected animal of animal species *P* will transmit disease *D* if in contact with susceptible livestock	Animal	Supplementary Table S3.1
*P*_*infa*_*n*__*CPD*__	Probability that product *P* from country *C* is derived from an animal infected with disease *D*	Product, Traveler	Eq. 7; Eq. 10
*P*_*con*_*t*__*PD*__	Probability that product *P* is contaminated with disease D	Product	Supplementary Table S3.5
*P*_*dets*_*l*__*D*__	Probability of detection of infection with disease *D* at slaughter	Product	Supplementary Table S3.4
*P*_*ex*_*p*__*P*__	Probability that a local animal is exposed to product *P*	Product	Supplementary Table S3.6
*P*_*conte*_*x*__*PD*__	Probability that product *P* is contaminated with disease *D* at exposure to a local animal	Product	Supplementary Tables S3.7; S3.8; S3.10
*P*_*infe*_*x*__*PD*__	Probability of infection of product *P* with disease *D* upon exposure to a local animal	Product	Supplementary Table S3.8
*Nt*_*C*_	Number of travelers arriving in the Netherlands from source country *C*	Traveler	(40)
*Pt*_*C*_	Fraction of travelers carrying products of animal origin when arriving from source country *C*	Traveler	(41–50)
*RP*_*CP*_	Probability that an animal product carried by a traveler arriving from source country *C* is of product type *P*	Traveler	(41–50, 52); Supplementary Table S2.3
*W*_*CP*_	Average weight (kg) of product type *P* carried per traveler arriving from source country *C*	Traveler	(52); Supplementary Table S2.3;
*P*_*h*_*m*__*P*__	Proportion of “homemade” product *P*	Traveler	(53)

The overall risk score R_*P*_ for a target area by a single introduction route *i* is calculated as:


(1) $$\begin{array}{r} {R_{P}}_{i}=1-\overset{c}{\underset{C=1}{\prod }}\overset{p}{\underset{P=1}{\prod }}\overset{d}{\underset{D=1}{\prod }}{\left(1-P_{entry_{CPD}}\times P_{est_{PD}}\right)}^{N_{CP}} \end{array}$$

where *N*_*CP*_ is the number of pathway units (animals for livestock, pets and exotic mammals; batches for poultry, exotic birds and germplasm; kg for animal products) of pathway *P* that enters the target area from source country *C* in the time period considered, *P*_*entr*_*y*__*CPD*__ is the probability of entry of disease *D* from source country *C* by pathway *P*, and *P*_*es*_*t*__*PD*__ is the probability that entry of disease *D* by pathway *P* results in a first local infection (establishment) in the target area. Although the overall risk score of RRAT is calculated as the probability of a successful introduction of any disease in the tool, it cannot be interpreted as such, because input into the tool is partly based on proxy values that were assigned to risk classes, rather than strictly quantitative data derived from e.g., scientific literature or animal experiments. Proxy values were defined as approximate values that represent the—sometimes unknown, and mostly uncertain – actual values of input parameters. The risk score is thus a semi-quantitative score, that can be calculated at different levels, e.g., per disease, source country or pathway, and can as such be used to rank diseases, source countries and pathways for their incursion risk.

Due to its asymptotic nature, the overall risk score *R*_*P*_ is not discriminating if its value approaches one for multiple diseases, source countries or pathways. Therefore, a second risk score indicating the number of successful introductions, *R*_*N*_, is calculated as:


(2) $$\begin{array}{r} {R_{N}}_{i}=\overset{c}{\underset{C=1}{\sum }}\overset{p}{\underset{P=1}{\sum }}\overset{d}{\underset{D=1}{\sum }}N_{CP}\times P_{entry_{CPD}}\times P_{est_{PD}} \end{array}$$

Again, although being calculated as the number of successful introductions of any disease in the tool, this risk score cannot be interpreted as such given its semi-quantitative nature.

### Trade of live animals

Data on the numbers and batches of animals traded to the Netherlands from each source country was derived from TRACES (23) (Supplementary Table S1.1). RRAT not only considers the trade in livestock, but also trade in equines, dogs, cats, and exotic mammals and birds. Animals were grouped based on species and destination (for life or for slaughter). This resulted in a total of 38 animal species groups (pathways) considered for this introduction route.

For each disease in RRAT, the animal species groups were assigned a susceptibility class based on information derived from factsheets and scientific literature (24–30). Five susceptibility classes were used: (1) reservoir host, (2) spill-over host possibly contributing to transmission (3) host in which only experimental infections have been described, (4) dead-end host, and (5) not susceptible (Supplementary Tables S2.1, S3.1).

**Figure 1:**
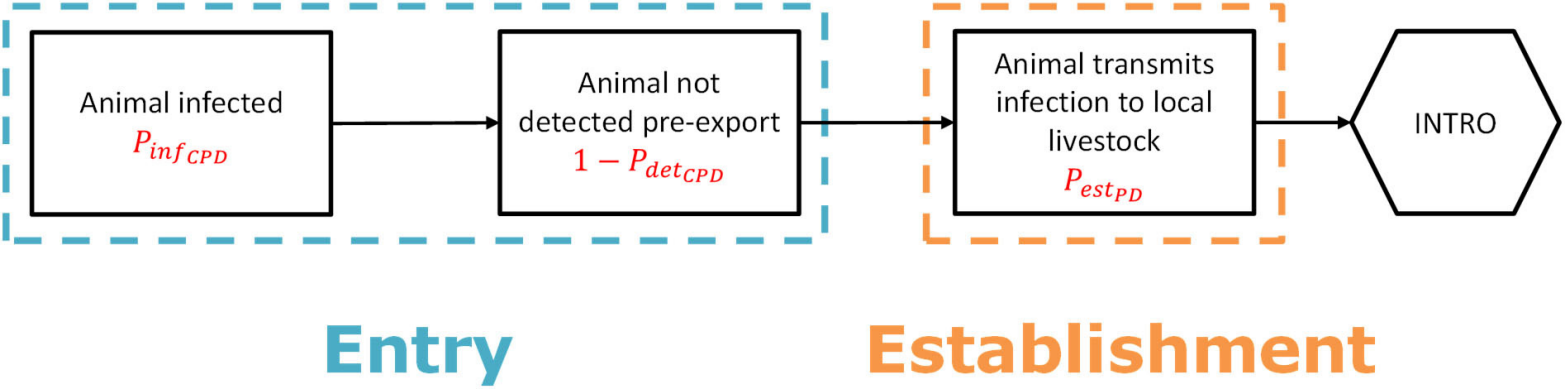
Scenario tree outlining the steps to assess the probability of entry and first infection for the legal trade in live animals (“animal route”).

To estimate the probability of entry (*P*_*entr*_*y*__*CPD*__), two main parameters were used: (1) the probability of an individual animal being infected (*P*_*in*_*f*__*CPD*__), and (2) the probability of an infected animal not being detected before transport (1−*P*_*de*_*t*__*CPD*__) (Figure 1). *P*_*entr*_*y*__*CPD*__ was calculated as:


(3) $$\begin{array}{r} P_{entry_{CPD}}=P_{inf_{CPD}}\times \left(1-P_{det_{CPD}}\right) \end{array}$$

*P*_*in*_*f*__*CPD*__ was estimated using data on disease incidence in the source countries and disease-specific parameters. *P*_*in*_*f*__*CPD*__ was calculated as:


(4) $$\begin{array}{r} P_{inf_{CPD}}=Inc_{CD}\times T_{inf_{D}}\times P_{infsus_{P}} \end{array}$$

where *Inc*_*CD*_ is the incidence of disease *D* in source country *C*, *P*_*infsu*_*s*__*P*__ is a proxy value to account for the probability of infection of animal species *P* with disease *D* dependent on its susceptibility class, and *T*_*in*_*f*__*D*__ is a proxy value to account for the average infectious period of disease *D* in reservoir hosts. The calculation of *Inc*_*CD*_ was based on all cases reported to the OIE in a one-year period (see Section “Disease incidence” for more details). However, for most diseases, animals are only infectious for a relatively short period. Therefore, *T*_*in*_*f*__*D*__ was used to correct for the average infectious period of infected animals, using four classes and accompanying proxy values (*T*_*in*_*f*__*D*__ = 0.05 if infectious period <2 weeks; *T*_*in*_*f*__*D*__ = 0.1 if infectious period > 2 weeks and <1 month; *T*_*in*_*f*__*D*__= 0.25 if infectious period > 1 month and <1 year; *T*_*in*_*f*__*D*__= 1 if infectious period > 1 year) (Supplementary Table S3.2). As *Inc*_*CD*_ was based on reported cases in reservoir livestock hosts only, *P*_*infsu*_*s*__*P*__ was used to correct for the expected incidence of disease in non-reservoir hosts with the value of *P*_*infsu*_*s*__*P*__ dependent on the animal's susceptibility class (Supplementary Table S3.1).

*P*_*de*_*t*__*CPD*__ was estimated using data on European legislation regarding both intracommunity trade (between European Union (EU) member states) and importation of animals from non-EU countries (31–38). Legal requirements such as clinical inspection, quarantine and testing, or importations from disease-free regions only were listed per disease, pathway (animal species) and source country. Individual measures were rated with a score between 0 and 1 for their effectiveness using information on e.g., length of the incubation period, severity of clinical signs, and test sensitivity. If > 1 measure was in force, *P*_*de*_*t*__*CPD*__ was set equal to the effectiveness of the most effective measure.

The probability of a first infection (*P*_*es*_*t*__*PD*__) was estimated considering the infectiousness and the destination of the imported animals. *P*_*es*_*t*__*PD*__ was calculated as:


(5) $$\begin{array}{r} P_{est_{PD}}=P_{contact_{P}}\times P_{trans_{PD}} \end{array}$$

where *P*_*contac*_*t*__*P*__ is a proxy value to account for the probability that the imported infected animal comes into contact with susceptible livestock in the target area, and *P*_*tran*_*s*__*PD*__ is a proxy value to account for the probability that the infected animal will transmit the disease if in contact with susceptible livestock. The value of *P*_*contac*_*t*__*P*__ depends on the destination of the imported animal (Supplementary Table S3.3). The value of *P*_*tran*_*s*__*PD*__ depends on the susceptibility class of the imported animal (Supplementry Table S3.1). Although the infection can be carried by dead-end hosts, they do not contribute to transmission of the disease and will as such not result in a successful introduction.

### Trade of animal products

Data on the import of live animal products (germplasm and hatching eggs) and manure was derived from TRACES (23), because TRACES provided most detail on the animal species from which these products were derived. Imports of germplasm (semen, embryos and ova) were, however, only available at batch level. Data on the import of other animal products was derived from Comext (20) (Supplementary Table S1.2). Animal products were assigned to animal product groups considering product type (meat, casings, milk and dairy products, eggs and egg products, hides, feathers and down, etc.), treatment (fresh, frozen, dried, salted, heated, etc.) and the animal species from which the product was derived (bovines, pigs, sheep, goats, equines, poultry, etc.). This resulted in a total of 139 pathways considered for this introduction route. For presentation purposes, results were aggregated in 16 summarizing product groups (Supplementary Table S2.2).

**Figure 2:**
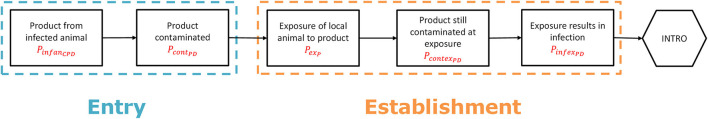
Scenario tree outlining the steps to assess the probability of entry and first infection for the legal trade of animal products including germplasm (“product route”).

To estimate the probability of entry (*P*_*entr*_*y*__*CPD*__), two main parameters were used: (1) the probability that the product is derived from an infected animal (*P*_*infa*_*n*__*CPD*__), and (2) the probability that the product itself is contaminated (*P*_*con*_*t*__*PD*__) (Figure 2). *P*_*entr*_*y*__*CPD*__ was calculated as:


(6) $$\begin{array}{r} P_{entry_{CPD}}=P_{infan_{CPD}}\times P_{cont_{PD}} \end{array}$$

*P*_*infa*_*n*__*CPD*__ was estimated taking into account the infection probability of the animal from which the product was derived in the source country (*P*_*in*_*f*__*CPD*__) and the probability of detection of the infection in the animal at ante-mortem or post-mortem inspection in the slaughterhouse (*P*_*dets*_*l*__*D*__, Supplementary Table S3.4). The latter only applied to products derived from slaughtered animals such as meat and hides, but not to products derived from live animals such as milk, eggs, germplasm and manure (Supplementray Table S2.2). To retrieve the value of *P*_*infa*_*n*__*CPD*__, the animal species from which the products were derived were linked to the 38 animal species groups used for the introduction route of live animal imports. A worst-case approach was applied here, i.e., if a product could have been derived from > 1 animal species, it was linked to all relevant animal species groups and given the value of *P*_*in*_*f*__*CPD*__ of the animal species group that was most susceptible to the disease. *P*_*infa*_*n*__*CPD*__ was calculated as:


(7) $$\begin{array}{r} P_{infan_{CPD}}=P_{inf_{CPD}}\times \left(1-P_{detsl_{D}}\right) \end{array}$$

*P*_*con*_*t*__*PD*__ was based on data derived from factsheets and scientific literature and entered into RRAT as an absence/presence score (Supplementary Figure S3.1, Supplementary Table S3.5).

To estimate the probability of first infection (*P*_*es*_*t*__*PD*__), three parameters were used: (1) the probability that a local animal is exposed to the product in the target area (*P*_*e*_*x*__*P*__), the probability that the product still contains viable pathogen when local animals are exposed to it (*P*_*conte*_*x*__*PD*__), and (3) the probability that such exposure results in infection (*P*_*infe*_*x*__*PD*__) (Figure 2). *P*_*es*_*t*__*PD*__ was calculated as:


(8) $$\begin{array}{r} P_{est_{PD}}=P_{ex_{P}}\times P_{contex_{PD}}\times P_{infex_{PD}} \end{array}$$

*P*_*e*_*x*__*P*__ is given by a proxy value accounting for the probability that the imported product will end up with local livestock animals and was made dependent on the intended use of the product (Supplementary Table S3.6). *P*_*conte*_*x*__*PD*__ depends on the survival time of the pathogen in the product and the average time it will take for the product to reach local animals, which is dependent on e.g., shipping time and shelf life. The latter is difficult to estimate and will probably be quite long for most products. *P*_*conte*_*x*__*PD*__ was therefore based on risk classes accounting for survival time and products were assigned to a risk class based on reported survival time in factsheets and literature (Supplementary Table S3.7, Supplementary Figure S3.1). The risk class was reduced by one level if import of the product from infected territories was subjected to import restrictions according to OIE (25) or EU legislation (39). Each risk class was given a probability score on a log_10_ scale to obtain proxy values for *P*_*conte*_*x*__*PD*__ (Supplementary Table S3.8). *P*_*infe*_*x*__*PD*__ was also given a proxy value based on risk classes, using the same log_10_ scale as for *P*_*conte*_*x*__*PD*__. The risk classes for this parameter were assigned considering the most likely exposure route to the pathogen dependent on its intended use (Supplementary Table S3.6). The probability that exposure results in infection depends on the exposure route and is disease specific, with some diseases more readily transmitted by e.g., aerosols whereas others are more readily transmitted by oral ingestion. The risk class assigned to each exposure route was therefore made disease-dependent (Supplementary Table S3.9).

### Animal products carried by air travelers

No database was available to directly input the amount of animal products carried by air travelers into RRAT. To estimate the volume of this introduction route, data on air passenger transport between the main airports of the Netherlands and their main partner airports (40) was combined with data from Great Britain on seizures of animal products (both meat and dairy products) (41–50) and input from scientific literature. Carrying animal products for own use into the Netherlands is illegal only if imported from non-EU countries. Therefore, customs do not search luggage of people traveling within the EU and hence no data was available to estimate the flow of products coming from EU member states. The incursion risk by this introduction route was thus evaluated for non-EU countries only.

Seizures of animal products were classified according to the type of product (meat; dairy; eggs), the animal species of which the product was derived (bovines; pigs; sheep; goats; poultry; buffalo; bushmeat), and the treatment of meat (fresh and frozen; dried and salted; heated). This resulted in a total of 21 pathways considered for this introduction route (Supplementary Table S2.3). No equine products were considered for the traveler route, and hence the incursion risk of the equine diseases AHS and EIA was not estimated for this introduction route. Data on seizures of animal products was not available at country level, but for 14 geographical regions comprising multiple countries (41) (Supplementary Figure S3.2). In RRAT, each source country was assigned to one of those 14 regions to extract the corresponding values from the database and calculations were performed at country level. Results of this introduction route are, however, presented at regional level, matching the lowest spatial resolution in the data.

The amount of animal products carried by people traveling to the Netherlands (*N*_*CP*_) was calculated as:


(9) $$\begin{array}{r} N_{CP}=Nt_{C}\times Pt_{C}\times RP_{CP}\times W_{CP} \end{array}$$

where *Nt*_*C*_ is the number of air travelers arriving in the Netherlands from source country *C* during a one-year period, *Pt*_*C*_ is the fraction of travelers carrying products of animal origin when arriving from source country *C*, *RP*_*CP*_ is the fraction of products carried by travelers from source country *C* that is of product type *P*, and *W*_*CP*_ is the average weight (kg) of product type *P* carried per traveler arriving from source country *C*. Note that values for *Pt*_*C*_, *RP*_*CP*_ and *W*_*CP*_ were only available at regional level (Supplementary Table S2.3). The calculated amounts are given in Supplementary Table S1.3.

The number of air travelers (*Nt*_*C*_) was derived from the Eurostat database table *avia_par_nl*, where the transport measurement (tra_meas) was *passengers carried – arrivals* (PAS_CRD_ARR) (40). This table reports on all passengers on a specific flight (with a single flight number) that terminate their journey at the reporting airport. Therefore, it was assumed that all passengers would have the Netherlands as destination (no transit passengers included). For journeys including multiple flights, the airport of embarkation was not known, resulting in an underestimate of the number of travelers arriving from non-EU countries.

Very little information was available to estimate the fraction of air travelers carrying products of animal origin (*Pt*_*C*_) and most estimates from literature were biased, i.e., passenger checks were risk-based, likely resulting in an overestimate of the probability that travelers will carry animal products. In RRAT, *Pt*_*C*_ was set to 15.5% based on estimates from Great Britain that 63.8% of the travelers that carry products of animal origin bring meat (43–50), and that 9.9% of all travelers bring meat (42). The value of 15.5% was used for all source regions. The RRAT is, however, flexible to include source region-specific values for this parameter. The probability that an animal product carried by a traveler arriving from source country *C* is of product type *P* (*RP*_*CP*_) was based on the proportion of seizures per product type from travelers arriving from the 14 different regions. Estimates for the proportions of meat from bovines, pigs, small ruminants, buffaloes and bushmeat were derived from VLA (42). These were complemented with data from Defra (43–50) to estimate the ratio of meat to dairy products. This ratio varied widely between regions with dairy constituting only 5% of seizures from Southern Africa and as much as 60% of seizures from Southern Asia (Supplementary Table S2.3). Estimates for the proportion of poultry in total meat were derived from scientific literature, with several publications reporting proportions of approximately 40% (51, 52). Eggs and egg products were estimated to be only 1% of animal products carried by travelers (51–55). Reported average weights per seizure (*W*_*CP*_) are mostly between 2 and 4 kg (42, 51, 53, 54, 56, 57), although seizures of bushmeat tend to have a higher weight than seizures of livestock meat (52, 57). In RRAT, region-based weights to estimate *W*_*CP*_ were derived from a study from Switzerland (52). No detail was available to estimate the weights for the different product types. The RRAT is, however, flexible to include product type-specific values for this parameter if new data would become available.

The calculations to estimate the probability of entry (*P*_*entr*_*y*__*CPD*__) and establishment (*P*_*es*_*t*__*PD*__) of pathogens via animal products carried by travelers were analogous to the calculations for the product route. Input parameters were derived by connecting each of the 21 pathways of the traveler route to one of the 139 pathways of the product route that had similar characteristics with respect to product type, treatment and animal species. Because it was assumed that part of the products carried by travelers were derived from animals slaughtered at home, an additional parameter (*P*_*h*_*m*__*P*__) was introduced to account for the fact that detection of infected animals at the slaughterhouse was less likely for products carried by travelers than for legally imported products. *P*_*h*_*m*__*P*__ is the proportion of products carried by travelers that is “homemade” and its value was set to 0.29 for all product types derived from dead livestock animals (53), and to 1 for bushmeat. The RRAT is, however, flexible to include product type-specific values for this parameter. The probability that the product carried by a traveler was derived from an infected animal (*P*_*infa*_*n*__*CPD*__) was therefore calculated as:


(10) $$\begin{array}{r} P_{infan_{CPD}}=P_{inf_{CPD}}\times \left(1-P_{detsl_{D}}\times \left(1-P_{hm_{P}}\right)\right) \end{array}$$

Products carried by travelers were assumed to have the same intended use as legally imported products, i.e., the probability of exposure to local animals is equal for both introduction routes. However, products carried by travelers escape import controls and therefore cannot be assessed for compliance with OIE standards or EU legislation. Therefore, the input values for the probability that the product still contains viable pathogen when local animals are exposed to it (*P*_*conte*_*x*__*PD*__) were separately estimated for this introduction route (Supplementary Table S3.10, Supplementary Figure S3.1).

### Disease incidence

**Figure 3:**
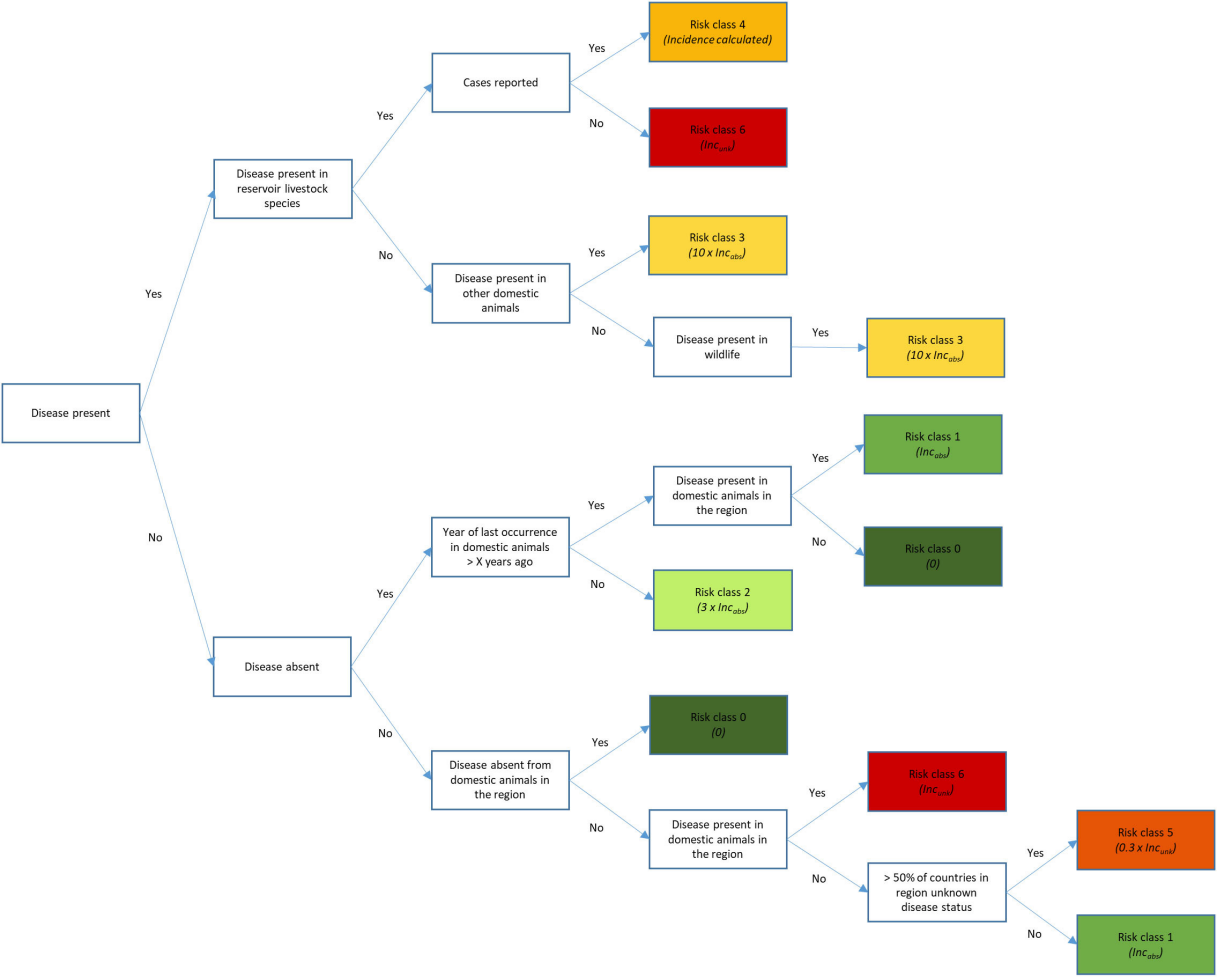
Decision tree to assign countries to one out of seven risk classes regarding disease incidence, considering the information available in the OIE annual reports (7).

Data on disease presence in the world was based on annual reports of individual countries in WAHIS (58). These reports were obtained using web scraping, because the data was not downloadable at the time RRAT was built. A decision tree was used to assign each country to a risk class considering the information the country had provided in the annual report to the OIE (Figure 3), and the reported disease incidence by countries in the same UN subregion (59). The decision tree distinguishes three main groups of countries: those that reported presence of disease in either wildlife or domestic animals (upper branch), those that reported absence of disease (middle branch), and those that had not provided any information on the disease for the year considered (lower branch). Only for countries that had reported cases to the OIE (risk class 4), the incidence of disease *D* in source country *C* (*Inc*_*CD*_) could be calculated by dividing the number of cases of disease *D* in livestock reservoir hosts in source country *C* (58) by the population of affected livestock reservoir hosts for disease *D* present in source country *C* (58, 60). For EU member states, the OIE data was complemented with data from the Animal Disease Information System (ADIS) (61) and reports from the European Commission (EC) on diseases in bovines and swine (62–64). These sources only provided the number of outbreaks, not the number of cases. The number of outbreaks was therefore multiplied with the median number of cases per outbreak as reported by WAHIS (58) (Supplementary Table S3.2) to arrive at an estimate of the number of cases in order to calculate *Inc*_*CD*_. If no data was available on the number of cases at all, a proxy value was used for *Inc*_*CD*_ based on the assigned risk class for disease presence. If a country had reported absence of disease, the year of last occurrence was considered for the risk classification, where we assumed that real absence was more likely if the disease had not been reported for a longer period (X years, where X was disease-dependent, Supplementary Table S3.2) and if the disease was not present in the UN subregion either. If no information was available on the disease status of a country, information on disease occurrence in the UN subregion was used to assign this country to a risk class. Only for countries assigned to risk class 0, *Inc*_*CD*_ was set to 0 as we deemed presence of the disease in those countries extremely unlikely. For the countries assigned to other risk classes, proxy values were used for *Inc*_*CD*_ that were either based on *Inc*_*ab*_*s*__*D*__ or *Inc*_*un*_*k*__*D*__. *Inc*_*ab*_*s*__*D*__ equaled an incidence 100 times lower than the minimum incidence for disease *D* calculated for countries in risk class 4 (disease present and cases reported), whereas *Inc*_*un*_*k*__*D*__ equaled the maximum incidence calculated for disease *D* for countries in risk class 4 (Supplementary Table S3.2).

### Baseline scenario

In the baseline scenario, model input as described above was used to estimate the incursion risk of 10 livestock diseases (AHS, ASF, Auj, BT, bTB, CSF, EIA, FMD, LSD, PPR) for the Netherlands for the years 2016, 2017, and 2018. Model calculations in RRAT are deterministic resulting in a point estimate for each output parameter. Main output parameters considered were the risk scores for individual diseases (_*R*_*P*_*i, D*_) for each introduction route per year, and the contribution of source countries and pathways to these estimated risk scores.

### Sensitivity analysis

Sensitivity analysis was performed to explore the impact of assumptions and input parameters on the results of RRAT. Three main areas of input uncertainty were investigated: (1) the incidence of disease in source countries, (2) the use of proxy values to estimate probabilities, and (3) the databases used to derive the volume of animals and animal products for the animal and traveler pathway, respectively (Table 3). A total of 12 alternative scenarios was run and results were compared to the baseline scenario for the overall risk score _*R*_*N*_*i*_ (Eq. 2) per introduction route. Considering that the main objective of RRAT is to prioritize diseases for risk management, changes in the overall risk score might be of less concern than changes in ranking of diseases, source countries, or pathways with respect to their incursion risk. To analyze the impact of uncertainty on ranking, the risk scores for individual diseases (_*R*_*N*_*i, D*_) and individual source countries (_*R*_*N*_*i, C*_) were ranked for both the baseline scenario and the alternative scenarios and compared using Spearman's rank correlation coefficient. The number-based risk score *R*_*N*_ was used for the sensitivity analysis rather than the probability-based risk score *R*_*P*_, because differences between scenarios cannot be observed for high probability-based scores due to the asymptotic nature of *R*_*P*_.

**Table 3:** Alternative scenarios explored in the sensitivity analysis to evaluate the impact of uncertain input parameters on the results of RRAT.

**No**.	**Scenario**	**Parameter**	**Baseline value**	**New value**	**References**
	*Incidence of disease*				
1A	Regions	Regions used to assign countries to risk classes for disease incidence	UN subregions	Adjusted UN subregions	(59, 76); Supplementary Figure S4.1
1B	Minimum incidence	Proxy value to estimate disease incidence for risk classes 1, 2 and 3 (*Inc*_*abs*_*D*__)	Value 100 times less than minimum incidence calculated for countries in risk class 4	Value equal to minimum incidence calculated for countries in risk class 4	Supplementary Table S3.2; S4.1
1C	Maximum incidence	Proxy value to estimate disease incidence for risk classes 5 and 6 (*Inc*_*unk*_*D*__)	Value equal to maximum incidence calculated for countries in risk class 4	Value of 0.1 or 0.3 dependent on disease characteristics such as incubation period, transmission rate, and clinical signs	Supplementary Table S3.2; Supplementary Table S4.1
1D	Scaling factor for risk classes	Multiplication factor to calculate disease incidence for risk classes 2, 3 and 5	risk class 2 = 3 × *Inc*_*abs*_*D*__; risk class 3 = 10 × *Inc*_*abs*_*D*__; risk class 5 = 0.3 × *Inc*_*unk*_*D*__	risk class 2 = 10 × *Inc*_*abs*_*D*__; risk class 3 = 100 × *Inc*_*abs*_*D*__; risk class 5 = 0.1 × *Inc*_*unk*_*D*__	Figure 3
1E	Underreporting	Underreporting factor	No underreporting assumed	Inclusion of an underreporting factor of 2.5 or 4 to calculate disease prevalence for countries in risk class 4; value dependent on disease characteristics such as incubation period, transmission rate, and clinical signs	(42, 76); Supplementary Table S4.1
	*Proxy values*	
2A	Probability infection	Probability of infection (_*P*_*infsus*_*P*_) for non-reservoir hosts	10^−2^ for spill over hosts; 10^−3^ for experimental hosts; 10^−2^ for dead end hosts	10^−3^ for spill over hosts; 10^−4^ for experimental hosts; 10^−3^ for dead end hosts	Supplementary Table S3.1
2B	Probability transmission	Probability of transmission (*P*_*trans*_*PD*__) for non-reservoir hosts	0.3 for spill over hosts; 0.1 for experimental hosts	0.1 for spill over hosts; 0.03 for experimental hosts	Supplementary Table S3.1
2C	Probability contact with susceptible livestock	Probability of contact (*P*_*contact*_*P*__) for all destinations but reservoir hosts going to livestock farms	10^−1^ for household, trade, approved body or livestock farm if non-reservoir host; 10^−2^ for slaughterhouse	10^−2^ for household, trade, approved body or livestock farm if non-reservoir host; 10^−3^ for slaughterhouse	Supplementary Table S3.3
2D	Probability product contaminated at exposure	Proxy value for the risk classes for the probability of contamination at exposure (*P*_*contex*_*PD*__)	high = 1; moderate = 0.1; low = 0.01; very low = 0.001	high = 1; moderate = 0.3; low = 0.1; very low = 0.03	Supplementary Table S3.8
2E	Probability infection upon exposure	Proxy value for the risk classes for the probability of infection upon exposure (*P*_*infex*_*PD*__)	high = 1; moderate = 0.1; low = 0.01; very low = 0.001	high = 1; moderate = 0.3; low = 0.1; very low = 0.03	Supplementary Table S3.8
	*Databases*	
3A	Eurostat (animals)	Number of imported live animals (*N*_*CP*_)	Data from TRACES	Data from Comext	(20, 23)
3B	PAS_BRD_ARR (travelers)	Number of travelers (*N*_*CP*_)	Data filtered for PAS_CRD_ARR (passengers carried – arrivals) in Eurostat database *avia_par_nl*	Data filtered for PAS_BRD_ARR (passengers on board – arrivals) in Eurostat database *avia_par_nl*	(40)

## Results

### Baseline scenario

**Figure 4:**
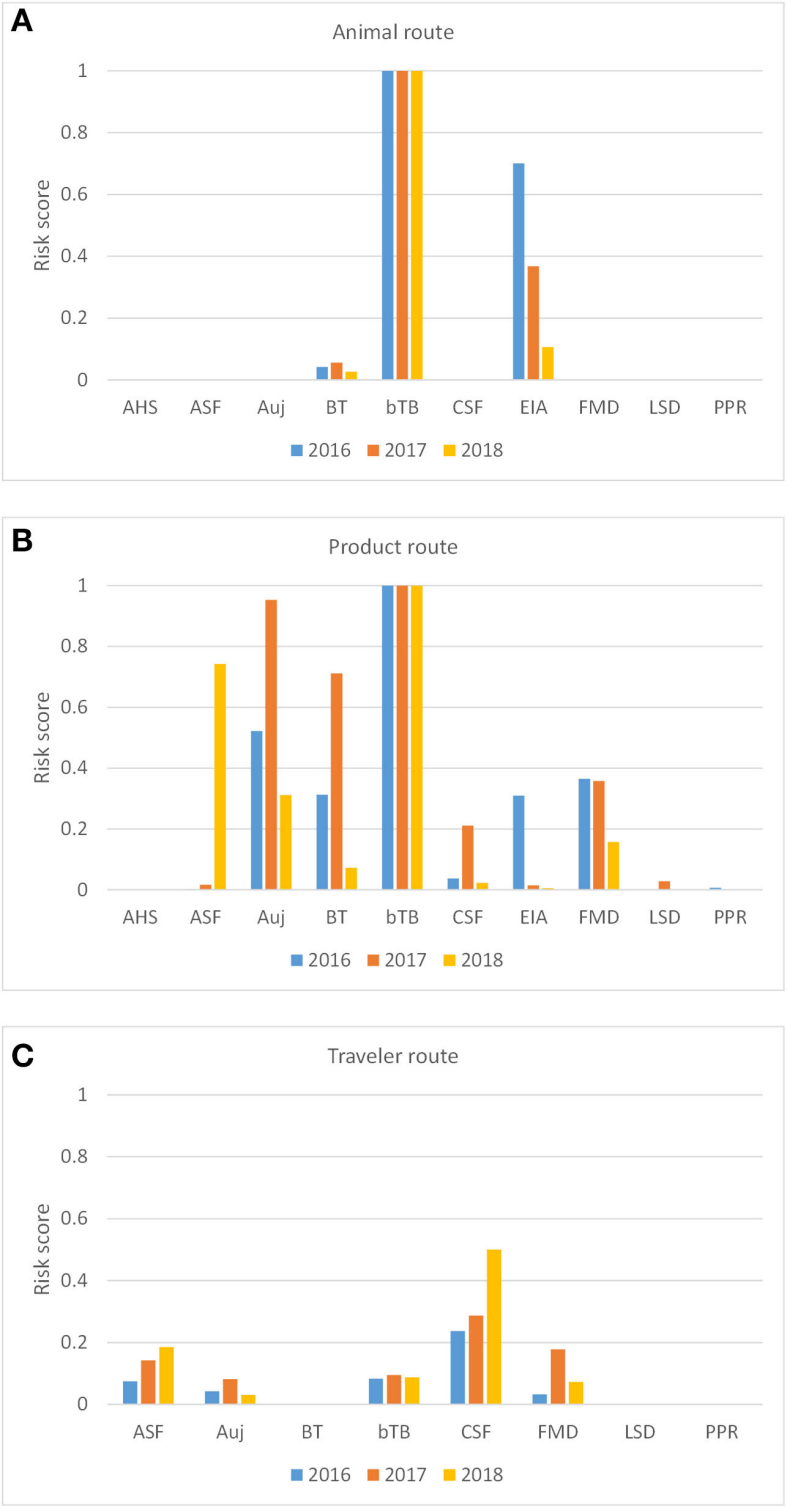
Probability-based risk score for the incursion risk of 10 diseases for the Netherlands in 2016, 2017, and 2018 for the animal route **(A)**, the product route **(B)**, and the traveler route **(C)**. The incursion risk of AHS and EIA were not considered for the traveler route. Diseases: AHS, African horse sickness; ASF, African swine fever; Auj, Aujeszky's disease; BT, bluetongue; bTB, bovine tuberculosis; CSF, classical swine fever; EIA, equine infectious anemia; FMD, foot-and-mouth disease; LSD, lumpy skin disease; PPR, peste des petits ruminants.

Model calculations returned a risk score (_*R*_*P*_*i, D*_) for each disease and each introduction route in RRAT for the Netherlands for the years 2016-2018 (Figure 4). The overall risk was highest for bTB with risk scores approaching 1 for both the animal route and the product route. The incursion risk of AHS, LSD and PPR, on the other hand, was very low for all introduction routes. Trade in live animals also posed a risk for EIA incursion, although the risk decreased over the years considered, and – to a much lesser extent – for BT incursion (Figure 4A). Despite the threatening ASF situation in Europe in the period 2016-2018, the probability of ASF incursion by the animal route was very low, because no live pigs were imported from infected countries. The relatively low incursion risk of most diseases for the animal route is explained by the fact that livestock animals were almost exclusively imported from European countries in which most of the diseases considered were reported absent. Trade of animal products entailed an incursion risk for a larger number of diseases than trade in live animals, since products were imported from a much wider geographical range including sometimes infected areas. The highest incursion risks for the product route were observed for bTB, Auj, BT and FMD. The incursion risk for ASF had increased tremendously in 2018 if compared to previous years which is explained by the expansion of ASF-infected territories in 2018, both in Europe and South-East Asia (6). Calculated risk scores for the traveler route were much lower than for the product route. Diseases most likely introduced *via* the traveler route were CSF, ASF, FMD and bTB. Although travelers are not allowed to carry animal products from outside the EU, products were carried from all over the world including regions from which legal import of products is restricted. This resulted in a different ranking of diseases for the traveler route than the product route.

**Figure 5:**
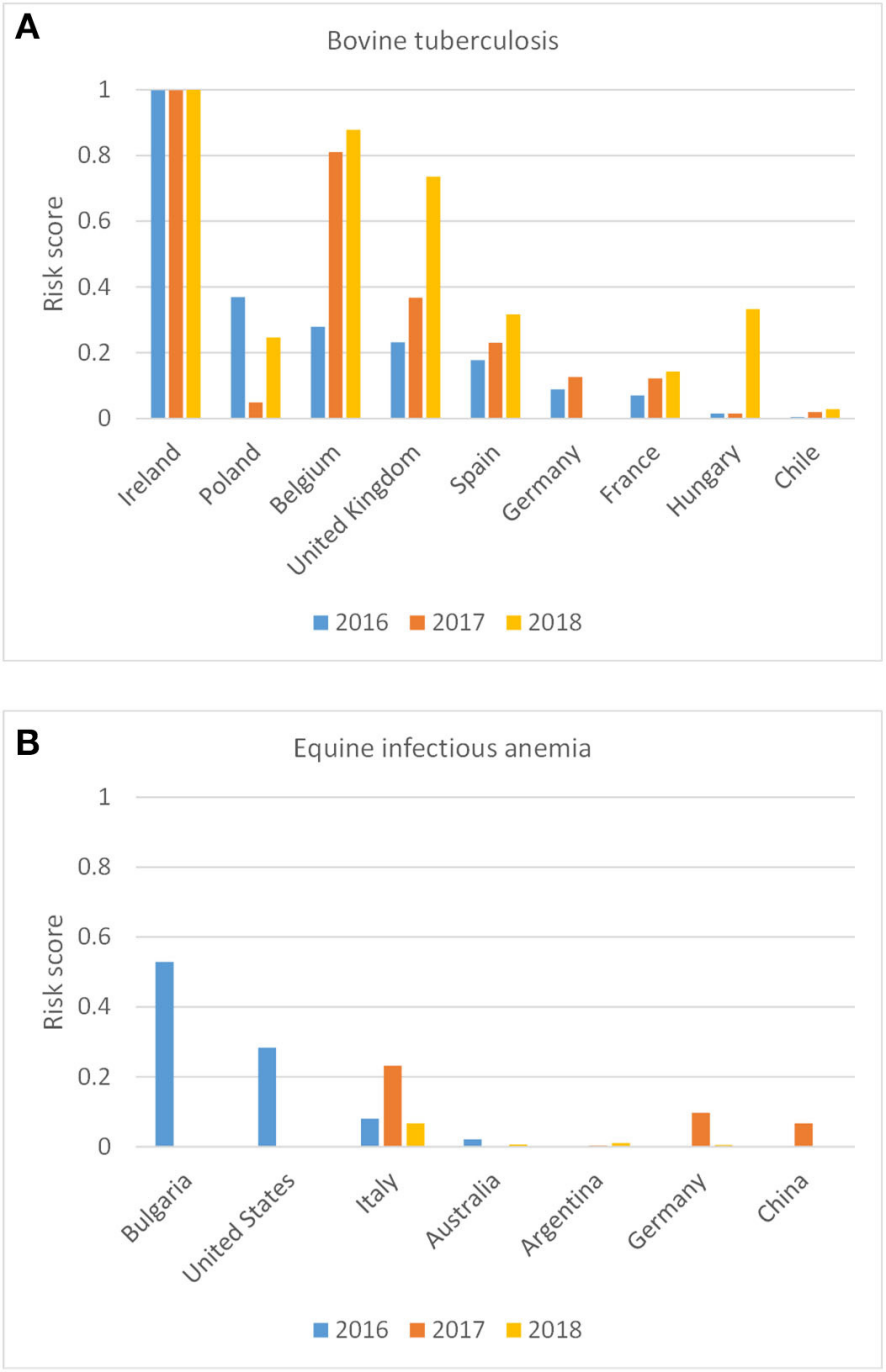
Probability-based risk score for the incursion risk of bovine tuberculosis **(A)** and equine infectious anemia **(B)** for the Netherlands in 2016, 2017, and 2018 for the animal route per source country (only source countries included with a risk score > 0.01 in any year).

As the RRAT calculates individual risk scores for each disease, pathway and source country, results can be explored in more detail to elucidate the countries and/or pathways contributing most to the incursion risk for a specific disease. Figure 5A shows the incursion risk of bovine tuberculosis per source country for the animal route and indicates that the incursion risk mainly originated from Ireland, Poland, Belgium, the United Kingdom and Spain. This was either related to a high incidence of bTB in those countries, high numbers of bovines imported from those countries (mainly veal calves), or both. Remarkably, there was also a risk of introducing bTB by trade of animals originating from Chile. This was related to the importation of camelids (lamas and vicunas). Horses entering the Netherlands more frequently originated from countries outside Europe than livestock animals. This is reflected by the countries contributing most to the incursion risk of EIA, not only being Bulgaria and Italy, but also the United States of America (Figure 5B).

**Figure 6:**
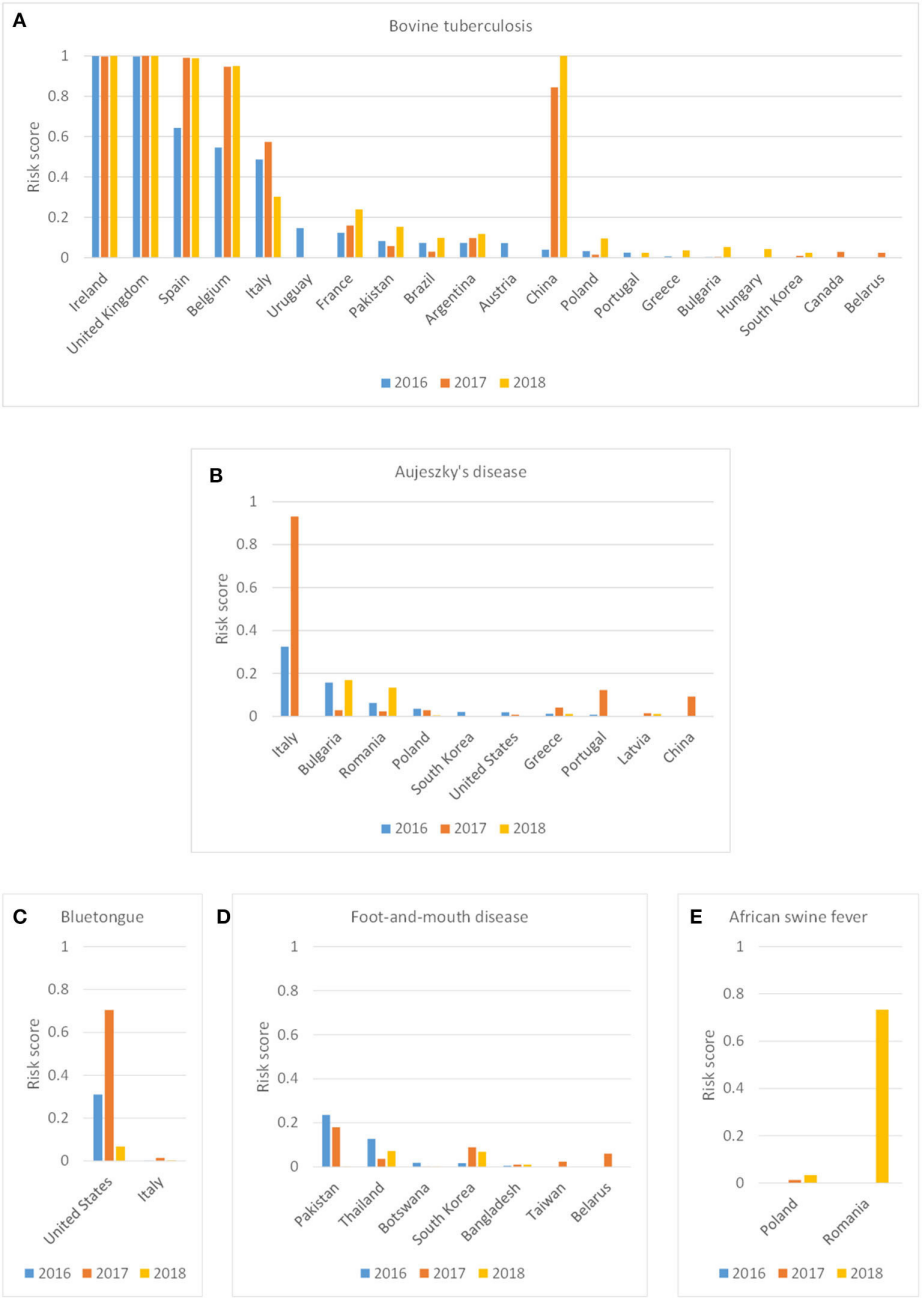
Probability-based risk score for the incursion risk of bovine tuberculosis **(A)**, Aujeszky's disease **(B)**, bluetongue **(C)**, foot-and-mouth disease **(D)** and African swine fever **(E)** for the Netherlands in 2016, 2017, and 2018 for the product route per source country (only source countries included with a risk score > 0.01 in any year).

**Figure 7:**
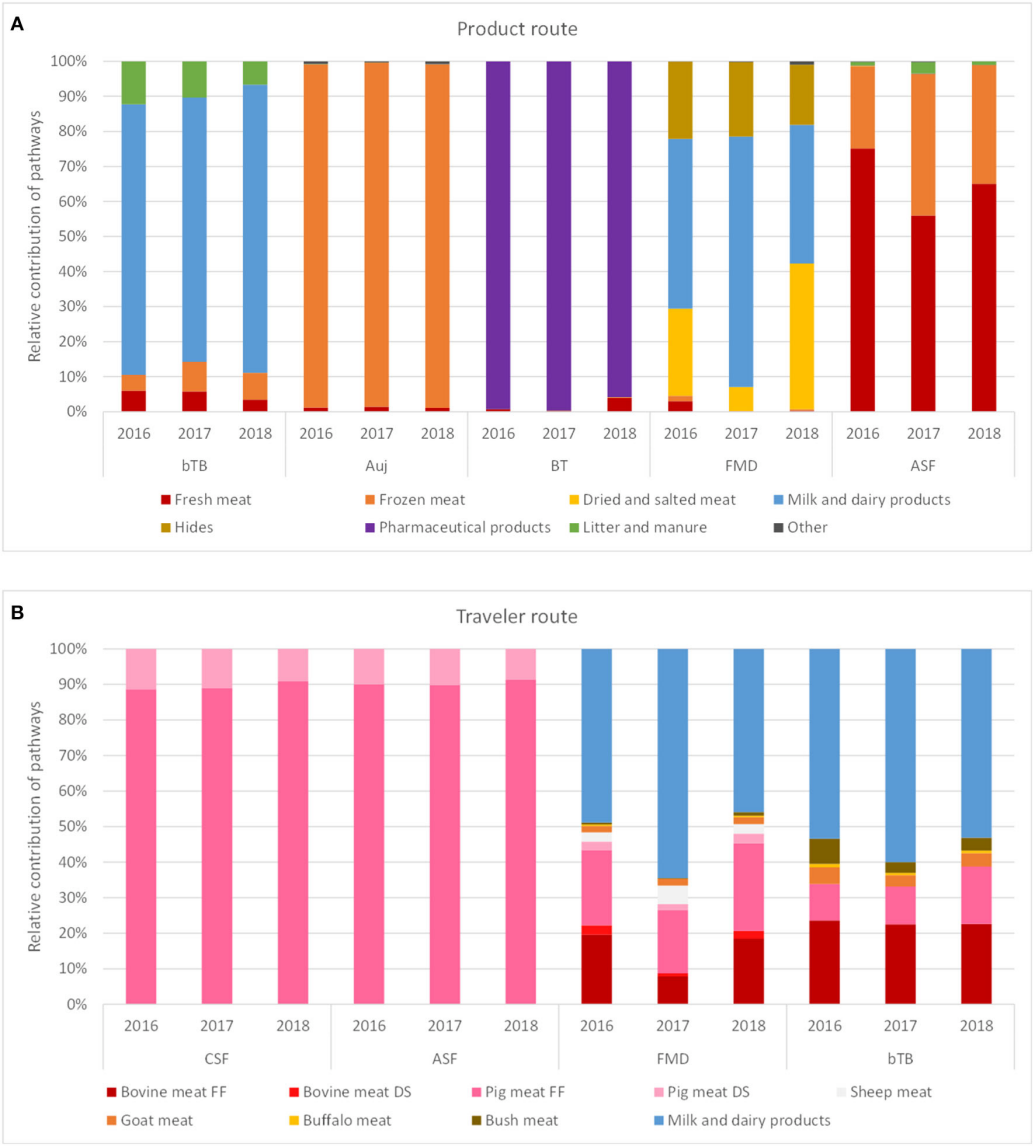
Contribution of different pathways to the incursion risk of selected diseases for the Netherlands in 2016, 2017, and 2018 for the product route **(A)** and the traveler route **(B)**. Diseases: bTB, bovine tuberculosis; Auj, Aujeszky's disease; BT, bluetongue; FMD, foot-and-mouth disease; ASF, African swine fever; CSF, classical swine fever. Products: FF, fresh and frozen meat; DS, dried and salted meat.

The incursion risk due to legal trade of animal products was explored in more detail for bTB, Auj, BT, FMD, and ASF. Whereas the incursion risk of bTB *via* the product route was high for multiple countries, with Ireland, United Kingdom, Spain, Belgium, Italy and China contributing most to the risk, the incursion risk of the other diseases was largely due to importations from a few countries only (Figure 6). The countries contributing most to the incursion risk for Auj were Italy, Bulgaria and Romania; for BT it was the USA; for FMD it were Pakistan, Thailand and South Korea; and for ASF it was Romania. Importations of meat products (fresh and frozen) contributed most to the incursion risk for Auj and ASF, whereas for bTB and FMD importations of milk and dairy products constituted a high incursion risk. The incursion risk of BT was mainly related to the import of products for pharmaceutical use (containing blood-derived products) (Figure 7A). It is noteworthy that import of litter and manure contributed considerably to the incursion risk of bTB and that import of hides contributed considerably to the incursion risk of FMD. Litter and manure were imported mainly from Belgium and Germany in large quantities (>2 × 10^3^ tons annually). This combined with bTB reported in Belgium resulted in a non-negligible incursion risk, albeit the probability of bTB infection of local animals upon entry of manure (*P*_*es*_*t*__*PD*__) was low. Hides were imported from all over the world with FMD-infected countries such as Thailand, India, Pakistan and South Korea being main suppliers from outside the EU. Although this contributed to the incursion risk of FMD, the overall incursion risk by hides was scored as low.

**Figure 8:**
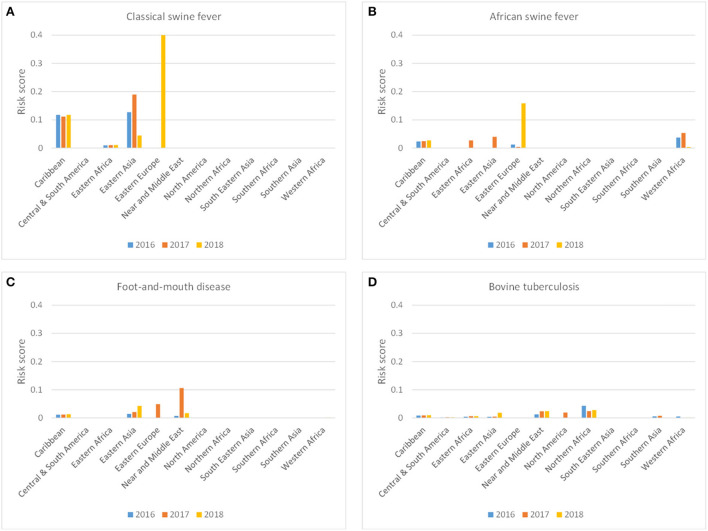
Probability-based risk score for the incursion risk of classical swine fever **(A)**, African swine fever **(B)**, foot-and-mouth disease **(C)** and bovine tuberculosis **(D)** for the Netherlands in 2016, 2017, and 2018 for the traveler route per source region.

Figure 8 shows the incursion risk due to travelers per source region for CSF, ASF, FMD, and bTB. CSF was most likely introduced from the Caribbean and Eastern Asia, although a steep increase in the incursion risk from Eastern Europe was observed in 2018. A similar risk profile was observed for ASF, although Western Africa was also a risk region for incursion of ASF *via* the traveler route. Overall risk scores for ASF were, however, lower than for CSF. For FMD, Eastern Asia and the Near and Middle East were the most likely source regions. bTB was most likely to be introduced from Northern Africa and the Near and Middle East. The incursion risk of CSF and ASF was completely related to carriage of pig meat, with fresh and frozen meat contributing approximately 90% to the risk and dried and salted meat approximately 10%. The incursion risk of FMD and bTB was related to both meat and dairy products, with dairy contributing approximately 50–60% of the risk (Figure 7B).

### Sensitivity analysis

**Figure 9:**
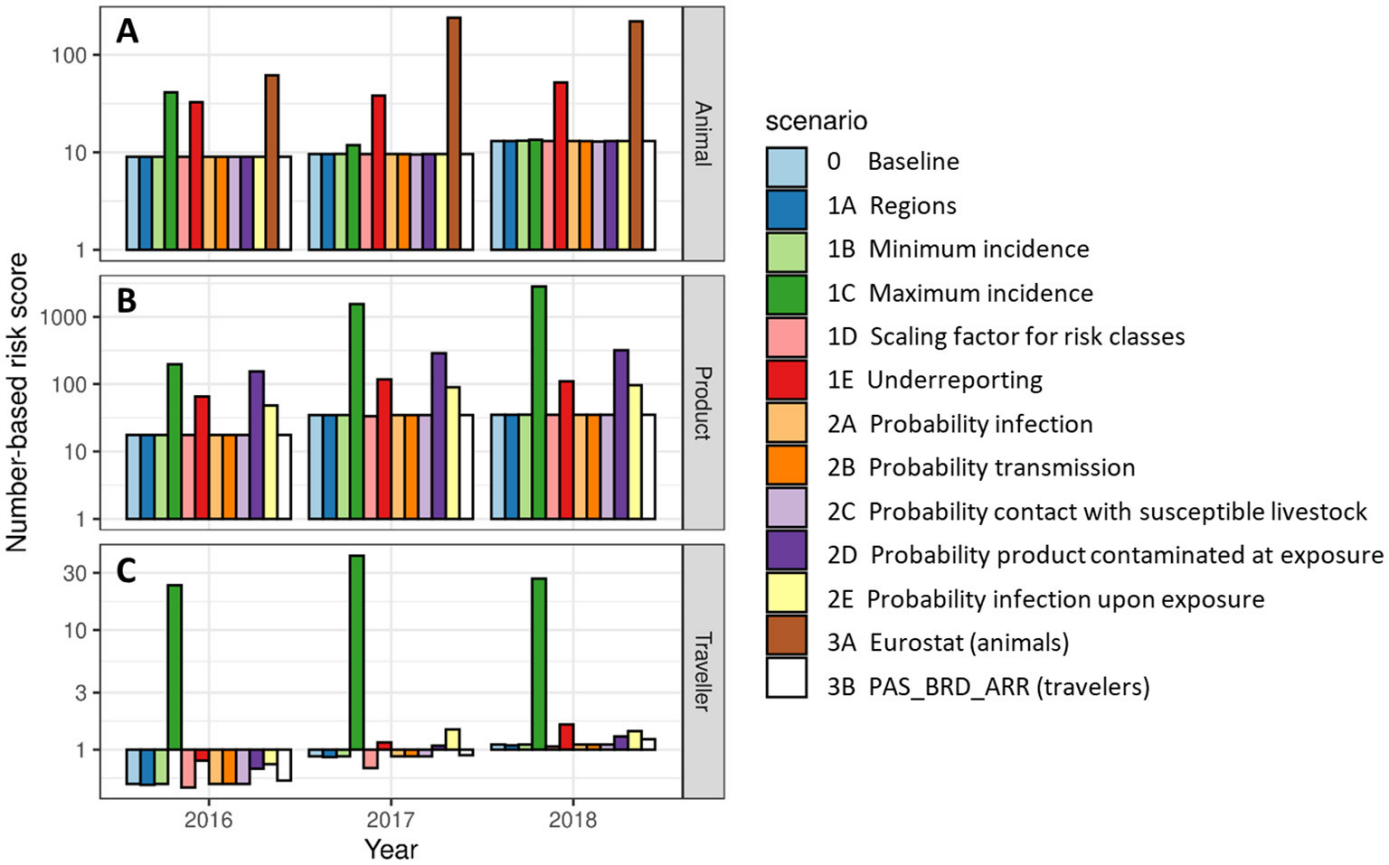
Number-based risk scores for the incursion risk of any disease for the Netherlands in 2016, 2017, and 2018 for the animal route **(A)**, the product route **(B)**, and the traveler route **(C)** for the baseline scenario and each alternative scenario. Risk scores are given on a log_10_ scale.

Results of the alternative scenarios (Table 3) were compared to the baseline scenario for the overall risk score _*R*_*N*_*i*_, which indicates the incursion risk of any of the diseases in RRAT to the Netherlands for each of the introduction routes *i* for the years 2016, 2017 and 2018. The number-based risk score was used rather than the probability-based risk score, as the probability-based risk has an asymptote at 1, making a comparison of results useless. The results of the animal route were most sensitive to the database used for the trade figures (Scenario 3A) with the use of Comext data (20) resulting in a 10-fold higher overall risk score (Figure 9). The other introduction routes were not affected by this scenario, since only the database for trade in live animals was changed. Scenario 1C affected the overall risk score most (Figure 9). In this scenario the value for *Inc*_*un*_*k*__*D*__ was increased 10- to 100-fold, resulting in a similar increase for the overall risk score of the product and traveler routes. The impact on the animal route was less pronounced, because imports of live animals mostly originated from source countries for which disease was absent or the incidence was known (i.e., *Inc*_*unk*_*D*__ was not needed to estimate disease incidence for these countries). Scenario 1E also affected the overall risk score of all three introduction routes, although to a lesser extent. In this scenario, an underreporting factor was included to estimate disease incidence for countries that had reported cases to the OIE, resulting in higher incidence estimates for these countries. Scenarios 2D (proxy values for probability of contamination of a product at exposure) and 2E (proxy values for probability of infection upon exposure to a contaminated product) resulted in an increased overall risk score for the product and traveler routes. This was not unexpected as higher proxy values were used in the alternative scenarios. These scenarios did not affect the animal route. All other scenarios had limited effect on the calculated overall risk scores.

**Figure 10:**
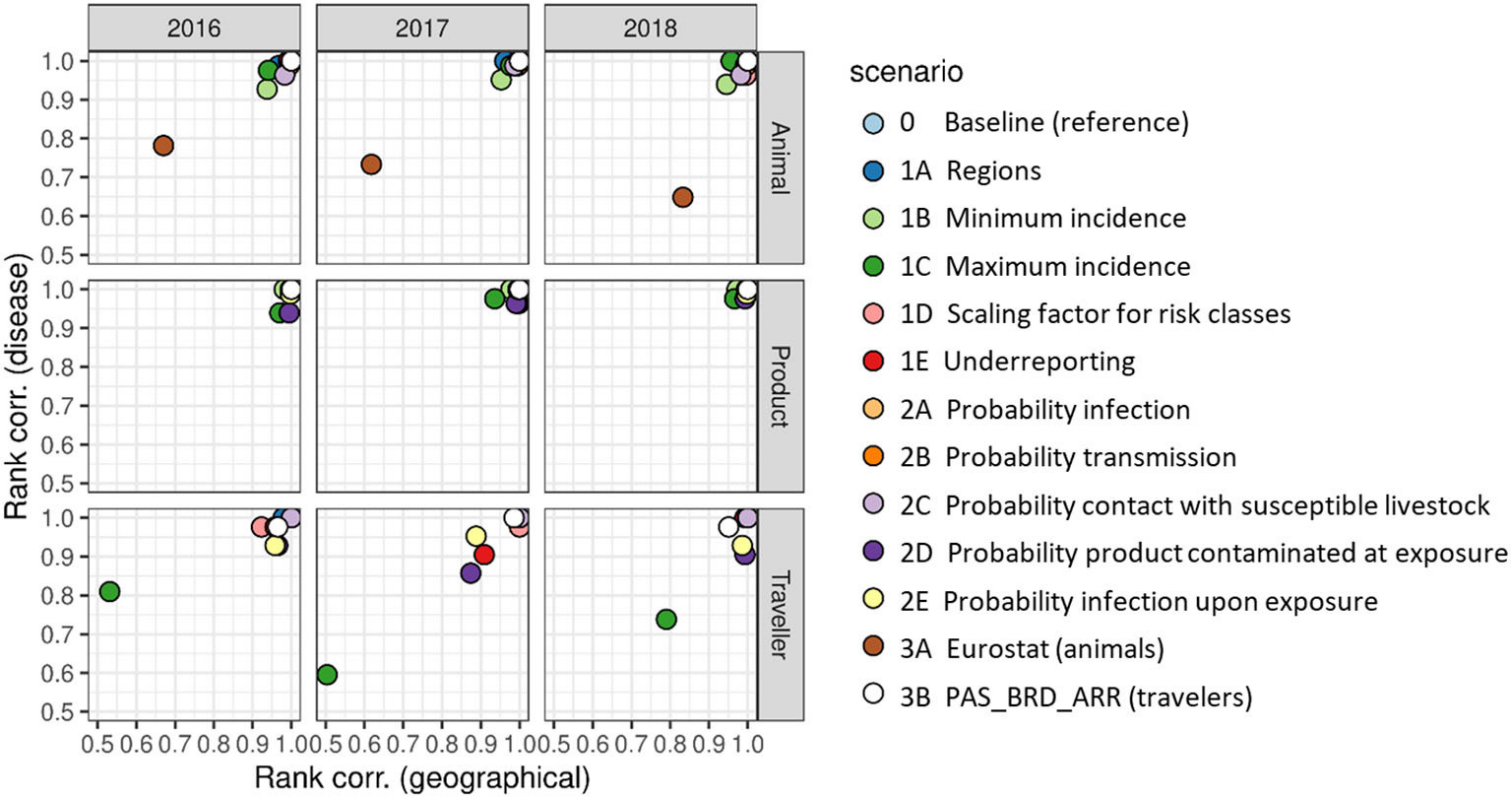
Spearman's rank correlation coefficients indicating the agreement in ranking of risk scores for individual source countries^a^ (x-axis, “geographical”) and individual diseases (y-axis, “disease”) between the baseline scenario and each alternative scenario. ^a^ Ranking for the traveler route was based on source regions.

Changes in ranking of diseases and source countries (source regions for the traveler route) when running the alternative scenarios were evaluated using Spearman's rank correlation coefficient. Figure 10 shows the correlation coefficients between the baseline and the alternative scenarios for the source countries/regions (x-axis) and the diseases (y-axis). Correlation coefficients for the product route were all > 0.9, indicating that changes in ranking were limited. For the animal route, only scenario 3A (trade figures based on Comext database) resulted in considerable changes of the ranking of both source countries and diseases for all 3 years evaluated. For the traveler route, results were slightly less stable than for the other two routes, but only scenario 1C (higher value for *Inc*_*un*_*k*__*D*__) resulted in considerable changes of the ranking of both source countries and diseases for all 3 years evaluated. The relative sensitivity of this route to the value of *Inc*_*un*_*k*__*D*__ is explained from the fact that travelers could come from any country in the world, including those countries with an unknown disease status, whereas imports of live animals and animal products were mostly limited to countries with a known disease status, although not exclusively.

## Discussion

### Interpretation of results

RRAT is a useful tool to assess the incursion risk of multiple diseases and results can be used to prioritize diseases for risk management and early warning. RRAT provides a multitude of information to evaluate the incursion risk of livestock diseases at different levels of detail. Results of the tool can be queried to indicate the pathways (animal species or product types) and source countries/regions contributing most to the risk (as shown in Figures 5–8). This information is useful input for the design of risk-based surveillance. To give risk managers access to all results of RRAT, an online visualization tool was built (https://shiny.wur.nl/content/941b9565-64d1-490c-b11b-d5f2cc45c44e/).

RRAT was built such that it can be automatically updated when new data becomes available. Input data from WAHIS (58), TRACES (23) and Comext (20) are automatically processed into risk scores for the diseases included in the tool. Adding a new disease to the tool is relatively easy as it only requires an update of disease-specific parameters. Adapting the tool to assess the risk for other target areas (countries) is also relatively easy, since this only requires an update of the volumes of trade and travel. The tool does, however, not provide the full remit of the incursion risk as introduction routes related to e.g., vector and wildlife ecology are not considered. This might explain the relatively low risk calculated for ASF despite the presence of ASF virus in Europe. Inclusion of additional introduction routes will increase the accuracy of the estimated incursion risk. Counotte et al. (65) designed a complementary module for RRAT using a generic approach to assess the animal disease incursion risk *via* wildlife migration. They showed that also the incursion risk of ASF *via* migration of wild boar is very low for the Netherlands given the relatively large geographical distance between reported outbreaks in wild boar and the Dutch border for the years analyzed. Results of the wildlife module can, however, not be directly compared to the results obtained for the introduction routes in RRAT, because the wildlife module only estimates the probability of entry of infected wild boar into the Netherlands and not the subsequent exposure of local livestock. It must be noted that also the results of the introduction routes in RRAT itself cannot be compared directly, as the risk estimates for the animal route are based on individual animals, whereas the risk estimates for the product and traveler route are based on kilograms of product. This might have resulted in a slight overestimate of the incursion risk by the product and traveler route, since from a single slaughtered animal more than one kilogram of product is harvested. There is no easy way to account for this in the risk assessment tool, as it is not known whether imported animal products are mostly derived from the same animals or from different animals, i.e., 10 kg of pork could have been derived from the same pig or from 10 pigs or even more. The ratio between slaughtered animals and the weight of imported products will probably also differ for different animal product types and source regions. To guide policy makers in interpreting the results of RRAT, we translated the semi-quantitative risk scores into qualitative risk levels. When doing so, we accounted for the fact that animal products will mostly present a lower incursion risk than live animals (Supplementary Table S5.1,S5.2). The qualitative risk levels were used to present results in the online visualization tool.

In contrast to the animal route, results of the product and traveler route can to some extent be compared, as both are based on kilograms of products. From Figure 4 it is clear that calculated risk scores for the traveler route are much lower than for the product route. This is mainly explained from the volumes for both introduction routes, with the quantities of products carried by travelers being approximately 10^3^ times less than the quantities imported legally (Supplementary Table S1.2,S1.3). On the other hand, products carried by travelers have a more diverse geographic origin and are not subjected to import controls, resulting in a potentially higher incursion risk per kilogram of product. The incursion risk *via* the product route might have been slightly overestimated by RRAT, as we had quite some uncertainty on the animal origin of products not intended for human consumption (e.g., casings, hides, products for pharmaceutical use). Most CN codes (combined nomenclature) (66) for these products represent composite groups and a worst-case approach was used considering all products a risk when these were derived from at least one susceptible domestic livestock species. Although products not intended for human consumption only made up about 10% of the total legal import flows, they had a very high contribution to the incursion risk of BT (Figure 7). In contrast to the product route, the incursion risk of the traveler route was based only on animal products for human consumption. The incursion risk *via* this route has definitely been underestimated by RRAT. We only included products carried by air passengers from outside the EU, since no data was available on products carried by travelers within the EU, because bringing products of animal origin from other EU member states is not illegal and thus not checked at customs. In addition, the incursion risk *via* animal products carried by travelers over land (train, bus, car) is not considered in RRAT.

### Validation of results

Validation of the results of RRAT is difficult as the tool estimates the incursion risk of diseases that are not introduced into the Netherlands regularly. The only exception is bTB for which 23 introductions occurred in the period 1999-2013 by trade in live animals (67). RRAT indeed indicated that trade in live animals entails a high risk of bTB introduction, indicating to a large extent the same source countries as high risk as the study of De Vos et al. (67). The estimated EIA incursion risk by legal trade in live animals was very high for 2016 and decreased in the years after. In 2017, the first (and until now only) case of EIA in the Netherlands was detected by serology, the moment of introduction of the infection being unknown (68). The estimated ASF incursion risk was relatively low, although a steep increase of the incursion risk by the product route was seen for 2018. Despite the increasing threat of ASF in Europe in recent years, at the time of writing (June 2022), ASF was absent from the Netherlands. The most likely introduction route for ASF, based on results of RRAT, is *via* legal trade of animal products. Although it cannot be excluded that contaminated pork products have been imported in recent years, this has not resulted in disease outbreaks. The probability that contaminated pork products end up with pigs is expected to be very low, as swill feeding is not allowed in the EU (69). The results of RRAT can also be partly validated by comparing the results of RRAT to those of bespoke RA models, although one should keep in mind that the risk estimates given by RRAT are semi-quantitative risk scores rather than absolute numbers. The incursion risk of AHS was, e.g., estimated to be very low by RRAT (Figure 4), which is in agreement with a quantitative risk assessment for movements of live equines by De Vos et al. (11).

RRAT was cross-validated against other generic risk assessment tools that recently were developed in Europe by applying all tools to the same case study on ASF (16). Results indicated that the generic tools largely agreed on the relative risks across countries and scenarios, resulting in the same ranking. RRAT was primarily designed for prioritization purposes, the ranking of diseases, source countries and pathways being thus the most important output of the tool. Therefore, the cross-validation contributed to the credibility of results obtained with RRAT. In addition, results for the years 2016-2018 were face validated by risk assessors and risk managers and any unexpected results were queried by investigating the underlying data in the tool. For instance, contrary to our expectations, China did not contribute much to the ASF incursion risk in 2018, despite presence of ASF in China since August 2018 (6, 70) and large volumes of pork products being imported from China (20). The huge pig population (4.3 × 10^8^ heads) (60) in China resulted, however, in a low estimate for the incidence of ASF and consequently also for the incursion risk posed by pig products imported from China.

### Robustness of results

RRAT can be classified as a semi-quantitative risk tool. The output of RRAT is presented as risk scores between 0 and 1. Although the risk score is calculated as if it were the probability of at least one introduction per year, the absolute value of the risk score cannot be interpreted as such, because input values for probabilities in RRAT are to a large extent based on risk classes rather than quantitative data derived from literature or experiments. These risk classes have been translated into proxy values to allow for the calculation of risk scores. Results of RRAT thus give an indication of relative risks rather than absolute risks and are therefore most useful for prioritization.

The impact of the proxy values was evaluated in the sensitivity analysis and appeared to be limited. In most scenarios, the change in proxy values did not affect the estimated risk scores. However, higher values for the probability of contamination of products at exposure, *P*_*contex*_*PD*__, and the probability of infection upon exposure, *P*_*infex*_*PD*__ (Figure 9; scenarios 2D and 2E), resulted in higher risk scores for the product and traveler route. The ranking of diseases, pathways and source countries/regions was, however, only slightly affected in these scenarios (Figure 10 and Supplementary Figure S4.2). Changing of the proxy values used to estimate the incidence of disease if countries had an unknown disease status (*Inc*_*unk*_*D*__) had a large impact on the estimated risk scores (Figure 9; scenario 1C). For the traveler route, the change of *Inc*_*unk*_*D*__ also had an impact on the ranking of diseases, pathways and source countries/regions (Figure 10 and Supplementary Figure S4.2).

Even though data from global databases is inputted into RRAT as purely quantitative data, these also contain uncertainty. Numbers of livestock imported, e.g., differ considerably between TRACES and Comext. The effect of using data from Comext (20) rather than TRACES was explored in scenario 3A. Results indicated that risk estimates based on Comext were much higher than based on TRACES (Figure 9). Ranking of diseases, pathways and source countries was also highly affected by the global database used (Figure 10 and Supplementary Figure S4.2). Similarly, data from WAHIS on disease occurrence worldwide is biased due to underreporting or non-reporting. Scenario 1E of the sensitivity analysis indicated that risk estimates were higher, especially for the animal route, when correcting for underreporting (Figure 9). Ranking of diseases, pathways and source countries/regions was, however, not affected (Figure 10 and Supplementary Figure S4.2). In this scenario we assumed equal underreporting for all geographic regions, whereas in reality there might be differences depending on, e.g., surveillance in place. Disease incidence could only be calculated for a subset of countries in which disease was present. Therefore, a decision tree was used in RRAT to classify countries for their disease risk based on quantitative and qualitative data available from WAHIS (Figure 3). If countries did not report at all (neither absence nor presence), they were classified as high risk, unless we had evidence that disease was likely to be absent based on information from other countries in the same region. For the EU, data on disease outbreaks from WAHIS was complemented with data from the Animal Disease Information System (ADIS) (61) and EC reports (62–64) if available. For countries in other regions in the world, the data in RRAT was solely derived from WAHIS. To account for the fact that disease might be present unnoticed, we also considered the disease status of neighboring countries (based on UN subregions) (59) to assign a disease status to countries that reported absence of disease. This sometimes resulted in a likely overestimate of the incursion risk, e.g., when considering the ASF incursion risk from Denmark, that is clustered with the Baltic states in which ASF has been present since 2014 (71).

Based on the results of the what-if analysis, we conclude that risk estimates given by RRAT are more sensitive to uncertainties in data reported by global databases than uncertainty introduced by expert opinion when using proxy values to assign quantitative probabilities to risk classes. Uncertainties in global databases can directly be traced to reporting issues, both when considering disease outbreaks and trade of animals and animal products. Where TRACES was built to track and trace animal movements within the EU from the perspective of animal and public health, the data in Comext is primarily obtained from import and export flows as declared by customs from an economic perspective.

We also calculated Spearman's rank correlation coefficients to compare the ranking of diseases, pathways and countries/regions among different years (Supplementary Figure S4.3). Strikingly, the differences between years were in general bigger than the differences observed between scenarios in the sensitivity analysis. This emphasizes that historical data cannot directly be used to predict future incursion risks. When we conceptualized RRAT, we aimed at regular updates of the risk assessments in an automated fashion to ensure that the estimated incursion risks reflect the current conditions. Therefore, RRAT has been designed such that updates of the assessment can be easily made when new data becomes available. RRAT is, however, dependent on data from global databases on disease outbreaks, and trade and travel, making the tool vulnerable to changes in these databases. In 2021, the OIE has launched a new WAHIS interface (7), making the R scripts that we prepared to scrape the annual reports off their website useless. This, and the delay in the launch and realization of the new WAHIS interface, has hampered timely updates of RRAT with 2019 and 2020 data. The next step in the development of RRAT is to adapt the R scripts such that we can easily import data on disease outbreaks from the new WAHIS website. The availability of application programming interfaces (api) to import data facilitates the use of global databases in estimating disease incursion risks. The development of generic risk assessment tools such as RRAT also illustrates the importance of building and maintaining global databases using the FAIR principles (findable, accessible, interoperable and reusable). Disease-specific parameters in RRAT have been entered once and are considered not to be subjected to change at short notice. The only exception is the legislation for import of live animals. EU requirements for importations of live animals have been regularly updated in recent years, especially for equines. Most changes had, however, little effect on the estimated incursion risks as they concerned source countries and animal species with low-volume trade flows. However, with the implementation of the Animal Health Law (72) in 2021, an update of the legislation tables in RRAT is needed. Unfortunately, we have not been able to design an automated procedure for this task.

### Comparison with other generic risk assessment tools

Several other generic risk assessment tools have been developed in recent years [e.g., (16, 17, 73–79)]. Each of these tools were developed with different objectives, and different approaches were used (16). Some of these tools can be used for rapid risk assessment in response to disease events and have expert opinion as input [e.g., (74, 75, 80)]. However, only few of these tools have, like RRAT, the data needed for the risk estimates available in the tool [e.g., (17, 76)], allowing for a rapid response without the need to bring disease experts together. The main asset of these tools is that risk assessments can be updated relatively easy, making the tools suitable for horizon scanning. Another difference is that some of the tools only address the probability of entry into a new area [e.g., (76)], whereas others also include epidemiological [e.g., (77)] or economic consequences [e.g., (75, 78, 79)]. RRAT has an in-between position by including the exposure assessment and the probability of a new infection, but not estimating subsequent spread of disease, or impact on animal health and economics. We deemed the inclusion of a first infection in local livestock a minimal requirement to make results of the tool meaningful, as import of contaminated products does not by definition result in disease outbreaks, nor does import of animals for slaughter or import of exotic animals in case of subclinical infections and no contacts with livestock farms. A shared challenge for these generic risk assessment tools is to keep them up and running and to have added value to policy makers in setting priorities for preventive measures and surveillance. Bianchini et al. (81) did a survey on the use of animal health information systems and risk analysis tools among professionals in animal and public health around the world. They concluded that the main areas of interest from these systems and tools are information on where diseases are present, pathways of introduction, and spread assessment. RRAT provides insight into the first two areas of interest. Results of RRAT are easily accessible *via* the online visualization tool, allowing for independent use by policy makers.

## Data availability statement

The original contributions presented in the study are included in the article/Supplementary material, further inquiries can be directed to the corresponding author.

## Author contributions

CV and MS initiated and conceptualized the research and drafted the equations and algorithms for RRAT. CV and RP designed the outline of RRAT. CV and RP collected data from global databases. RP cleaned and analyzed all data inputted into RRAT. CV, MS, and EK collected data on disease-specific parameters, legislation for animal trade and travelers. RP built the online visualization tool. CV analyzed the results of RRAT and drafted the manuscript. All authors contributed to the article and approved the submitted version.

## Funding

The development of RRAT was funded by the Dutch Ministry of Agriculture, Nature and Food Quality (KB-21-006-028, KB-37-003-033, WOT-01-003-078, and WOT-01-003-094) and Wageningen University & Research (KB-33-001-008-WBVR).

## Conflict of interest

The authors declare that the research was conducted in the absence of any commercial or financial relationships that could be construed as a potential conflict of interest.

## Publisher's note

All claims expressed in this article are solely those of the authors and do not necessarily represent those of their affiliated organizations, or those of the publisher, the editors and the reviewers. Any product that may be evaluated in this article, or claim that may be made by its manufacturer, is not guaranteed or endorsed by the publisher.
