# Supplementary material for: Rapid risk assessment tool (RRAT) to prioritize emerging and re-emerging livestock diseases for risk management
Source: Front Vet Sci. 2022 Sep 7;9:963758. doi: 10.3389/fvets.2022.963758 (PMC9490411; doi:10.3389/fvets.2022.963758)
Supplement: Supplementary file 1 [file Table_1.DOCX]

Supplementary Material 1: Description of pathway data used in RRAT

**Table S1.1.** Number of pathway units (animals for livestock and other mammals; consignments for poultry and other birds) transported to the Netherlands for each pathway (animal species group) considered for the animal introduction route. The numbers include both trade between EU member states and imports from non-EU member states. Source: TRACES, Netherlands Food and Consumer Product Safety Authority (NVWA).

| **Animal species group** | **Category** | **2016** | **2017** | **2018** |
| --- | --- | --- | --- | --- |
| Live horses, asses, mules, hinnies destined for life | Equines | 12858 | 14410 | 14040 |
| Live horses, asses, mules, hinnies destined for slaughter | Equines | -- | 1 | -- |
| Live bovines destined for life | Bovines | 800791 | 827234 | 873786 |
| Live bovines destined for slaughter | Bovines | 58006 | 43295 | 48100 |
| Live swine destined for life | Swine | 73478 | 73321 | 66062 |
| Live swine destined for slaughter | Swine | 482261 | 565660 | 254551 |
| Sheep destined for life | Small ruminants | 44800 | 44065 | 45408 |
| Sheep destined for slaughter | Small ruminants | 12662 | 10283 | 8248 |
| Goats destined for life | Small ruminants | 348 | 186 | 120 |
| Goats destined for slaughter | Small ruminants | 1608 | 1099 | 721 |
| Chickens (gallus domesticus) destined for life | Poultry | 2246 | 2200 | 2174 |

**Table S1.1.** Continued.

| **Animal species group** | **Category** | **2016** | **2017** | **2018** |
| --- | --- | --- | --- | --- |
| Chickens (gallus domesticus) destined for slaughter | Poultry | 45826 | 48266 | 45308 |
| Turkeys destined for life | Poultry | 75 | 68 | 77 |
| Turkeys destined for slaughter | Poultry | -- | -- | -- |
| Ducks destined for life | Poultry | 17 | 21 | 24 |
| Ducks destined for slaughter | Poultry | 512 | 453 | 440 |
| Geese destined for life | Poultry | 29 | 22 | 33 |
| Geese destined for slaughter | Poultry | -- | 2 | -- |
| Guinea fowls destined for life | Poultry | 2 | 5 | 8 |
| Guinea fowls destined for slaughter | Poultry | -- | -- | -- |
| Primates | Other mammals | 509 | 542 | 800 |
| Sea mammals | Other mammals | 12 | 2 | 6 |
| Camelidae | Other mammals | 207 | 408 | 429 |
| Rabbits and hares | Other mammals | 5 | 31 | 316 |
| Dogs | Other mammals | 11883 | 12232 | 12980 |
| Cats | Other mammals | 1839 | 1788 | 2183 |

**Table S1.1.** Continued.

| **Animal species group** | **Category** | **2016** | **2017** | **2018** |
| --- | --- | --- | --- | --- |
| Ferrets | Other mammals | 315 | 512 | 430 |
| Carnivora other than dogs, cats, ferrets | Other mammals | 125 | 664 | 623 |
| Artiodactyla | Other mammals | 105 | 207 | 182 |
| Perrisodactyla | Other mammals | 30 | 37 | 38 |
| Rodentia | Other mammals | 296186 | 148669 | 181610 |
| Other mammals | Other mammals | 37749 | 74284 | 22322 |
| Birds of prey | Other birds | 32 | 29 | 26 |
| Psittaciformes | Other birds | 405 | 709 | 577 |
| Ostriches and emus destined for life | Other birds | 6 | 12 | 2 |
| Ostriches and emus destined for slaughter | Other birds | -- | -- | 1 |
| Pigeons | Other birds | 37 | 51 | 63 |
| Other birds | Other birds | 182 | 113 | 135 |

AHS=African horse sickness; ASF=African swine fever; Auj=Aujeszky’s disease; BT=bluetongue; bTB=bovine tuberculosis; CSF=classical swine fever; EIA=equine infectious anemia; FMD=foot-and-mouth disease; LSD=lumpy skin disease; PPR=peste des petits ruminants

**Table S1.2.** Number of pathway units (consignments for germplasm; kg for animal products) transported to the Netherlands for each pathway (animal product group) considered for the product introduction route. The numbers include both trade between EU member states and imports from non-EU member states. Products that did not constitute an incursion risk for any of the diseases in RRAT have been omitted from the table (poultry products; heated meat products; products for industrial use; blood-meal, meat-and-bone meal, offal). Source: TRACES, Netherlands Food and Consumer Product Safety Authority (NVWA) (germplasm and manure); Eurostat, 2022a (other product groups).

| **Summarizing product group** | **Product** | **Animal species** | **2016** | **2017** | **2018** |
| --- | --- | --- | --- | --- | --- |
| Germplasm | Semen | Equines | 2311 | 3693 | 4326 |
| Germplasm | Embryos | Equines | -- | 11 | 14 |
| Germplasm | Semen | Bovines | 1169 | 1114 | 1097 |
| Germplasm | Embryos | Bovines | 133 | 137 | 174 |
| Germplasm | Semen | Pigs | 1724 | 1432 | 1171 |
| Germplasm | Embryos | Pigs | -- | -- | -- |
| Germplasm | Semen | Sheep | 1 | 2 | 1 |
| Germplasm | Embryos | Sheep | -- | -- | -- |
| Germplasm | Semen | Goats | 2 | 2 | 2 |
| Germplasm | Embryos | Goats | -- | -- | -- |
| Germplasm | Semen | Other mammals | 1 | 3 | 1 |
| Germplasm | Embryos | Other mammals | -- | 1 | -- |

**Table S1.2.** Continued.

| **Summarizing product group** | **Product** | **Animal species** | **2016** | **2017** | **2018** |
| --- | --- | --- | --- | --- | --- |
| Fresh meat | Fresh meat deboned | Equines | 5624716 | 5355363 | 5174864 |
| Fresh meat | Fresh meat on the bone incl. carcasses | Bovines | 239596123 | 244679620 | 244018134 |
| Fresh meat | Fresh meat deboned | Bovines | 117365266 | 114696611 | 115647976 |
| Fresh meat | Fresh meat on the bone incl. carcasses | Pigs | 122256377 | 166718426 | 112996752 |
| Fresh meat | Fresh meat deboned | Pigs | 108639601 | 131516129 | 128032506 |
| Fresh meat | Fresh or frozen meat deboned or dried and salted meat | Pigs | 69959628 | 70996107 | 68433954 |
| Fresh meat | Fresh meat on the bone incl. carcasses | Sheep | 9893345 | 12157992 | 14987931 |
| Fresh meat | Fresh meat deboned | Sheep | 6009346 | 7481255 | 6799284 |
| Fresh meat | Fresh meat on the bone incl. carcasses | Goats | 34 | 149 | 68 |

**Table S1.2.** Continued.

| **Summarizing product group** | **Product** | **Animal species** | **2016** | **2017** | **2018** |
| --- | --- | --- | --- | --- | --- |
| Fresh meat | Fresh meat deboned | Goats | 605 | 21451 | 455 |
| Fresh meat | Fresh meat deboned | Sheep/goats | 51207 | 76326 | 157 |
| Fresh meat | Fresh meat deboned | Equines/sheep/goats | 2640 | -- | -- |
| Fresh meat | Fresh or frozen meat deboned | Artiodactyla | 2902 | 22590 | 2403 |
| Fresh meat | Fresh or frozen meat deboned | Camelidae | 16522 | 111042 | 100414 |
| Fresh meat | Fresh meat deboned | Livestock mammals (undefined) | 1977703 | 2308397 | 2225096 |
| Fresh meat | Fresh meat deboned | Other mammals | 218482 | 382689 | 247484 |
| Fresh meat | Fresh or frozen meat deboned | Other mammals | 19045255 | 22907669 | 27378340 |
| Frozen meat | Frozen meat deboned | Equines | 2811640 | 6074438 | 9397502 |
| Frozen meat | Frozen meat on the bone incl. carcasses | Bovines | 5962362 | 5878852 | 10521367 |

**Table S1.2.** Continued.

| **Summarizing product group** | **Product** | **Animal species** | **2016** | **2017** | **2018** |
| --- | --- | --- | --- | --- | --- |
| Frozen meat | Frozen meat deboned | Bovines | 54791315 | 65605135 | 63902480 |
| Frozen meat | Frozen meat on the bone incl. carcasses | Pigs | 18791931 | 27267845 | 16875075 |
| Frozen meat | Frozen meat deboned | Pigs | 73053810 | 72764473 | 68273521 |
| Frozen meat | Frozen meat on the bone incl. carcasses | Sheep | 5112818 | 6905085 | 6085847 |
| Frozen meat | Frozen meat deboned | Sheep | 12613065 | 13062770 | 12814357 |
| Frozen meat | Frozen meat on the bone incl. carcasses | Goats | 39868 | 39513 | 35753 |
| Frozen meat | Frozen meat deboned | Goats | 22361 | 13679 | 26714 |
| Frozen meat | Frozen meat deboned | Sheep/goats | 2980366 | 2058015 | 2450352 |
| Frozen meat | Frozen meat deboned | Livestock mammals (undefined) | -- | -- | 1581299 |

**Table S1.2.** Continued.

| **Summarizing product group** | **Product** | **Animal species** | **2016** | **2017** | **2018** |
| --- | --- | --- | --- | --- | --- |
| Dried and salted meat | Dried and salted meat | Bovines | 1399167 | 488144 | 147048 |
| Dried and salted meat | Dried and salted meat | Pigs | 10930346 | 9395395 | 9431491 |
| Dried and salted meat | Dried and salted meat | Sheep/goats | 484 | 2709 | 669 |
| Dried and salted meat | Dried and salted meat | Artiodactyla | -- | -- | 15 |
| Dried and salted meat | Dried and salted meat | Livestock mammals (undefined) | 8089203 | 11196960 | 14307128 |
| Dried and salted meat | Dried and salted meat | Other mammals | 177720686 | 141225905 | 121483570 |
| Milk/dairy products^a^ | Milk/dairy products | Bovines/sheep/goats | 2426890518 | 2578072335 | 2689446164 |
| Milk/dairy products^b^ | Milk/dairy products | Bovines/sheep/goats | 67033129 | 79550587 | 93988968 |
| Casings | Casings | Livestock mammals (undefined) | 58740476 | 69733798 | 66751282 |
| Hides | Raw hides and skins | Equines/bovines | 83098990 | 94588343 | 75573896 |

**Table S1.2.** Continued.

| **Summarizing product group** | **Product** | **Animal species** | **2016** | **2017** | **2018** |
| --- | --- | --- | --- | --- | --- |
| Hides | Semi-processed hides and skins (usual chemical and mechanical processes in tanning industry) | Equines/bovines | 694407 | 780794 | 766642 |
| Hides | Wool (chemical treatment, or storage at specific temperature) | Equines/other mammals | 6752 | 6098 | 3332 |
| Hides | Raw hides and skins | Bovines | 2620768 | 3567945 | 3598347 |
| Hides | Semi-processed hides and skins (usual chemical and mechanical processes in tanning industry) | Bovines | 4709260 | 4994485 | 4571701 |
| Hides | Wool, hair (not treated) | Pigs | 1061281 | 729000 | 217415 |
| Hides | Raw hides and skins | Pigs | 371005 | 471028 | 368381 |
| Hides | Semi-processed hides and skins (usual chemical and mechanical processes in tanning industry) | Pigs | 3143499 | 1273736 | 118609 |
| Hides | Raw hides and skins | Sheep | 2596457 | 5547796 | 2398277 |

**Table S1.2.** Continued.

| **Summarizing product group** | **Product** | **Animal species** | **2016** | **2017** | **2018** |
| --- | --- | --- | --- | --- | --- |
| Hides | Semi-processed hides and skins (usual chemical and mechanical processes in tanning industry) | Sheep | 10548 | 3130 | 4874 |
| Hides | Wool, hair (not treated) | Sheep | 1574305 | 4355698 | 923429 |
| Hides | Wool (chemical treatment, or storage at specific temperature) | Sheep | 1356840 | 1460516 | 1021161 |
| Hides | Raw hides and skins | Goats | 2962 | 15410 | 1698 |
| Hides | Semi-processed hides and skins (usual chemical and mechanical processes in tanning industry) | Goats | 93662 | 83164 | 23117 |
| Hides | Wool, hair (not treated) | Camelidae/goats | 15 | 113 | 185 |
| Hides | Wool, hair (not treated) | Sheep/goats/camelidae/other mammals | 1368970 | 2212366 | 1863445 |
| Hides | Wool (chemical treatment, or storage at specific temperature) | Sheep/goats/camelidae/other mammals | 1172873 | 1401022 | 1972481 |
| Hides | Wool, hair (not treated) | Other mammals | 234693 | 416538 | 173679 |

**Table S1.2.** Continued.

| **Summarizing product group** | **Product** | **Animal species** | **2016** | **2017** | **2018** |
| --- | --- | --- | --- | --- | --- |
| Hides | Raw hides and skins | Other mammals | 395989 | 676978 | 463734 |
| Hides | Semi-processed hides and skins (usual chemical and mechanical processes in tanning industry) | Other mammals | 124278 | 170210 | 57863 |
| Hides | Raw hides and skins | Livestock mammals (undefined) | 1151456 | 882678 | 1245062 |
| Hides | Semi-processed hides and skins (usual chemical and mechanical processes in tanning industry) | Livestock mammals (undefined) | 489816 | 592300 | 586138 |
| Bones | Fresh bones | Livestock mammals (undefined) | 134978280 | 144381238 | 150623989 |
| Bones | Dried or disinfected bones | Livestock mammals (undefined) | 304 | NA | 3 |
| Bones | Fresh bones | Other mammals | 78017 | 80168 | 34903 |
| Bones | Dried or disinfected bones | Other mammals | 213945 | 246966 | 224157 |
| Bones | Bones | All live animals | 2162277 | 2125016 | 2075246 |

**Table S1.2.** Continued.

| **Summarizing product group** | **Product** | **Animal species** | **2016** | **2017** | **2018** |
| --- | --- | --- | --- | --- | --- |
| Pharmaceutical use | Pharmaceutical use | All live animals | 1903431 | 2683016 | 907704 |
| Litter and manure | Manure | Bovines | 41242501 | 45378465 | 49205810 |
| Litter and manure | Manure | Pigs | 168415141 | 178702463 | 162856358 |
| Litter and manure | Manure | Poultry (undefined) | 37767560 | 39158057 | 41861040 |
| Litter and manure | Manure | All live animals | 1339020 | 22000 | 44000 |

# ^a^ Milk and dairy products for human consumption

^b^ Milk and dairy products as an ingredient of animal feed

**Table S1.3.** Estimated amount of products (kg) illegally carried into the Netherlands by travelers for each pathway (animal product group) considered for the traveler introduction route. Products that did not constitute an incursion risk for any of the diseases in RRAT have been omitted from the table (poultry products; heated meat products). Data have been calculated using data on the number of travelers (Eurostat, 2022b) and data from Table S2.3.

| **Animal product group** | **2016** | **2017** | **2018** |
| --- | --- | --- | --- |
| FFM_bovine | 659700 | 702846 | 759887 |
| DSM_bovine | 106630 | 113539 | 123357 |
| FFM_swine | 491321 | 523148 | 543631 |
| DSM_swine | 62887 | 66844 | 70419 |
| FFM_sheep | 100292 | 105550 | 114237 |
| DSM_sheep | 11250 | 11922 | 13057 |
| FFM_goat | 52857 | 56393 | 60746 |
| DSM_goat | 4924 | 5278 | 5740 |
| FFM_buffalo | 11766 | 12780 | 14138 |
| DSM_buffalo | 1622 | 1756 | 1956 |
| FFM_bushmeat | 65621 | 76473 | 74747 |
| DSM_bushmeat | 6304 | 7237 | 7255 |
| Milk & dairy products | 1692639 | 1802578 | 1956823 |

# References

Eurostat. Comext Bulk Download. (2022a). Available online at: https://ec.europa.eu/eurostat/estat-navtree-portlet-prod/BulkDownloadListing?sort=1&dir=comext (accessed March 16, 2022).

Eurostat. European Statistics on Transport. (2022b). https://ec.europa.eu/eurostat/web/transport/data/database (accessed April 11, 2022).
