# Supplementary material for: Rapid risk assessment tool (RRAT) to prioritize emerging and re-emerging livestock diseases for risk management
Source: Front Vet Sci. 2022 Sep 7;9:963758. doi: 10.3389/fvets.2022.963758 (PMC9490411; doi:10.3389/fvets.2022.963758)
Supplement: Supplementary file 2 [file Table_2.docx]

Supplementary Material 2: Pathways included in RRAT

**Table S2.1.** Pathways (animal species groups) considered for the animal introduction route and their susceptibility to the 10 diseases in RRAT. Susceptibility classes: 0: not susceptible; 1: reservoir host; 2: spill over host possibly contributing to transmission; 3: host in which only experimental infections have been described; 4: dead-end host. Scores were based on data derived from OIE (OIE, 2021a; OIE, 2021b; OIE, 2022), factsheets (Dórea et al., 2017; CFSPH, 2022; Discontools, 2022; EFSA, 2022) and a review of scientific literature.

| **Animal species group** | **Category** | **AHS** | **ASF** | **Auj** | **EIA** | **BT** | **bTB** | **CSF** | **FMD** | **LSD** | **PPR** |
| --- | --- | --- | --- | --- | --- | --- | --- | --- | --- | --- | --- |
| Live horses, asses, mules, hinnies destined for life | Equines | 1 | 0 | 3 | 1 | 0 | 2 | 0 | 0 | 0 | 0 |
| Live horses, asses, mules, hinnies destined for slaughter | Equines | 1 | 0 | 3 | 1 | 0 | 2 | 0 | 0 | 0 | 0 |
| Live bovines destined for life | Bovines | 0 | 0 | 4 | 0 | 1 | 1 | 3 | 1 | 1 | 4 |
| Live bovines destined for slaughter | Bovines | 0 | 0 | 4 | 0 | 1 | 1 | 3 | 1 | 1 | 4 |
| Live swine destined for life | Swine | 0 | 1 | 1 | 0 | 0 | 1 | 1 | 1 | 0 | 4 |
| Live swine destined for slaughter | Swine | 0 | 1 | 1 | 0 | 0 | 1 | 1 | 1 | 0 | 4 |
| Sheep destined for life | Small ruminants | 0 | 0 | 4 | 0 | 1 | 2 | 3 | 1 | 3 | 1 |
| Sheep destined for slaughter | Small ruminants | 0 | 0 | 4 | 0 | 1 | 2 | 3 | 1 | 3 | 1 |
| Goats destined for life | Small ruminants | 0 | 0 | 4 | 0 | 1 | 1 | 3 | 1 | 3 | 1 |
| Goats destined for slaughter | Small ruminants | 0 | 0 | 4 | 0 | 1 | 1 | 3 | 1 | 3 | 1 |
| Chickens (gallus domesticus) destined for life | Poultry | 0 | 0 | 3 | 0 | 0 | 0 | 0 | 0 | 0 | 0 |

**Table S2.1.** Continued.

| **Animal species group** | **Category** | **AHS** | **ASF** | **Auj** | **EIA** | **BT** | **bTB** | **CSF** | **FMD** | **LSD** | **PPR** |
| --- | --- | --- | --- | --- | --- | --- | --- | --- | --- | --- | --- |
| Chickens (gallus domesticus) destined for slaughter | Poultry | 0 | 0 | 3 | 0 | 0 | 0 | 0 | 0 | 0 | 0 |
| Turkeys destined for life | Poultry | 0 | 0 | 3 | 0 | 0 | 0 | 0 | 0 | 0 | 0 |
| Turkeys destined for slaughter | Poultry | 0 | 0 | 3 | 0 | 0 | 0 | 0 | 0 | 0 | 0 |
| Ducks destined for life | Poultry | 0 | 0 | 3 | 0 | 0 | 0 | 0 | 0 | 0 | 0 |
| Ducks destined for slaughter | Poultry | 0 | 0 | 3 | 0 | 0 | 0 | 0 | 0 | 0 | 0 |
| Geese destined for life | Poultry | 0 | 0 | 3 | 0 | 0 | 0 | 0 | 0 | 0 | 0 |
| Geese destined for slaughter | Poultry | 0 | 0 | 3 | 0 | 0 | 0 | 0 | 0 | 0 | 0 |
| Guinea fowls destined for life | Poultry | 0 | 0 | 0 | 0 | 0 | 0 | 0 | 0 | 0 | 0 |
| Guinea fowls destined for slaughter | Poultry | 0 | 0 | 0 | 0 | 0 | 0 | 0 | 0 | 0 | 0 |
| Primates | Other mammals | 0 | 0 | 3 | 0 | 0 | 2 | 0 | 0 | 0 | 0 |
| Sea mammals | Other mammals | 0 | 0 | 0 | 0 | 0 | 2 | 0 | 0 | 0 | 0 |
| Camelidae | Other mammals | 2 | 0 | 0 | 0 | 1 | 1 | 0 | 2 | 0 | 2 |
| Rabbits and hares | Other mammals | 0 | 0 | 4 | 0 | 0 | 2 | 3 | 3 | 0 | 0 |
| Dogs | Other mammals | 2 | 0 | 4 | 0 | 2 | 2 | 0 | 0 | 0 | 2 |
| Cats | Other mammals | 0 | 0 | 4 | 2 | 2 | 0 | 0 | 0 | 0 | 0 |

**Table S2.1.** Continued.

| **Animal species group** | **Category** | **AHS** | **ASF** | **Auj** | **BT** | **bTB** | **CSF** | **EIA** | **FMD** | **LSD** | **PPR** |
| --- | --- | --- | --- | --- | --- | --- | --- | --- | --- | --- | --- |
| Ferrets | Other mammals | 0 | 0 | 4 | 0 | 2 | 0 | 0 | 0 | 0 | 0 |
| Carnivora other than dogs, cats, ferrets | Other mammals | 0 | 0 | 4 | 2 | 2 | 0 | 0 | 2 | 0 | 0 |
| Artiodactyla | Other mammals | 0 | 2 | 4 | 1 | 1 | 2 | 0 | 1 | 2 | 2 |
| Perrisodactyla | Other mammals | 2 | 0 | 0 | 2 | 2 | 0 | 0 | 2 | 0 | 0 |
| Rodentia | Other mammals | 0 | 0 | 4 | 0 | 2 | 0 | 0 | 3 | 0 | 0 |
| Other mammals | Other mammals | 0 | 0 | 4 | 0 | 2 | 0 | 0 | 3 | 0 | 0 |
| Birds of prey | Other birds | 0 | 0 | 0 | 0 | 0 | 0 | 0 | 0 | 0 | 0 |
| Psittaciformes | Other birds | 0 | 0 | 0 | 0 | 2 | 0 | 0 | 0 | 0 | 0 |
| Ostriches and emus destined for life | Other birds | 0 | 0 | 0 | 0 | 0 | 0 | 0 | 0 | 0 | 0 |
| Ostriches and emus destined for slaughter | Other birds | 0 | 0 | 0 | 0 | 0 | 0 | 0 | 0 | 0 | 0 |
| Pigeons | Other birds | 0 | 0 | 3 | 0 | 3 | 0 | 0 | 0 | 0 | 0 |
| Other birds | Other birds | 0 | 0 | 0 | 0 | 3 | 0 | 0 | 0 | 0 | 0 |

AHS=African horse sickness; ASF=African swine fever; Auj=Aujeszky’s disease; BT=bluetongue; bTB=bovine tuberculosis; CSF=classical swine fever; EIA=equine infectious anemia; FMD=foot-and-mouth disease; LSD=lumpy skin disease; PPR=peste des petits ruminants

**Table S2.2.** Pathways (animal product groups) considered for the product introduction route, their assumed destination, if derived from slaughtered animals yes or no, and the global database used. Sources: TRACES (EC, 2022); Comext (Eurostat, 2022). CN codes (combined nomenclature; EU, 1987) used for each pathway are given in Supplementary Material 6.

| **Summarizing product group** | **Product** | **Animal species** | **Destination** | **Slaughtered animal** | **Database** |
| --- | --- | --- | --- | --- | --- |
| Germplasm | Semen | Equines | Breeding | No | TRACES |
| Germplasm | Embryos | Equines | Breeding | No | TRACES |
| Germplasm | Semen | Bovines | Breeding | No | TRACES |
| Germplasm | Embryos | Bovines | Breeding | No | TRACES |
| Germplasm | Semen | Pigs | Breeding | No | TRACES |
| Germplasm | Embryos | Pigs | Breeding | No | TRACES |
| Germplasm | Semen | Sheep | Breeding | No | TRACES |
| Germplasm | Embryos | Sheep | Breeding | No | TRACES |
| Germplasm | Semen | Goats | Breeding | No | TRACES |
| Germplasm | Embryos | Goats | Breeding | No | TRACES |
| Germplasm | Semen | Other mammals | Breeding | No | TRACES |
| Germplasm | Embryos | Other mammals | Breeding | No | TRACES |
| Hatching eggs | Hatching eggs | Chicken | Breeding | No | TRACES |
| Hatching eggs | Hatching eggs | Poultry other than chicken | Breeding | No | TRACES |

**Table S2.2.** Continued.

| **Summarizing product group** | **Product** | **Animal species** | **Destination** | **Slaughtered animal** | **Database** |
| --- | --- | --- | --- | --- | --- |
| Hatching eggs | Hatching eggs | Birds other than poultry | Breeding | No | TRACES |
| Fresh meat | Fresh meat deboned | Equines | Human consumption | Yes | Comext |
| Fresh meat | Fresh meat on the bone incl. carcasses | Bovines | Human consumption | Yes | Comext |
| Fresh meat | Fresh meat deboned | Bovines | Human consumption | Yes | Comext |
| Fresh meat | Fresh meat on the bone incl. carcasses | Pigs | Human consumption | Yes | Comext |
| Fresh meat | Fresh meat deboned | Pigs | Human consumption | Yes | Comext |
| Fresh meat | Fresh or frozen meat deboned or dried and salted meat | Pigs | Human consumption | Yes | Comext |
| Fresh meat | Fresh meat on the bone incl. carcasses | Sheep | Human consumption | Yes | Comext |
| Fresh meat | Fresh meat deboned | Sheep | Human consumption | Yes | Comext |
| Fresh meat | Fresh meat on the bone incl. carcasses | Goats | Human consumption | Yes | Comext |

**Table S2.2.** Continued.

| **Summarizing product group** | **Product** | **Animal species** | **Destination** | **Slaughtered animal** | **Database** |
| --- | --- | --- | --- | --- | --- |
| Fresh meat | Fresh meat deboned | Goats | Human consumption | Yes | Comext |
| Fresh meat | Fresh meat deboned | Sheep/goats | Human consumption | Yes | Comext |
| Fresh meat | Fresh meat deboned | Equines/sheep/goats | Pharmaceutical use | Yes | Comext |
| Fresh meat | Fresh or frozen meat deboned | Artiodactyla | Human consumption | Yes | Comext |
| Fresh meat | Fresh or frozen meat deboned | Camelidae | Human consumption | Yes | Comext |
| Fresh meat | Fresh meat deboned | Livestock mammals (undefined) | Human consumption | Yes | Comext |
| Fresh meat | Fresh meat deboned | Other mammals | Human consumption | Yes | Comext |
| Fresh meat | Fresh or frozen meat deboned | Other mammals | Human consumption | Yes | Comext |
| Fresh meat | Fresh meat on the bone incl. carcasses | Chicken | Human consumption | Yes | Comext |

**Table S2.2.** Continued.

| **Summarizing product group** | **Product** | **Animal species** | **Destination** | **Slaughtered animal** | **Database** |
| --- | --- | --- | --- | --- | --- |
| Fresh meat | Fresh meat deboned | Chicken | Human consumption | Yes | Comext |
| Fresh meat | Fresh meat on the bone incl. carcasses | Turkey | Human consumption | Yes | Comext |
| Fresh meat | Fresh meat deboned | Turkey | Human consumption | Yes | Comext |
| Fresh meat | Fresh meat on the bone incl. carcasses | Ducks | Human consumption | Yes | Comext |
| Fresh meat | Fresh meat deboned | Ducks | Human consumption | Yes | Comext |
| Fresh meat | Fresh meat on the bone incl. carcasses | Geese | Human consumption | Yes | Comext |
| Fresh meat | Fresh meat deboned | Geese | Human consumption | Yes | Comext |
| Fresh meat | Fresh meat on the bone incl. carcasses | Guinea fowl | Human consumption | Yes | Comext |

**Table S2.2.** Continued.

| **Summarizing product group** | **Product** | **Animal species** | **Destination** | **Slaughtered animal** | **Database** |
| --- | --- | --- | --- | --- | --- |
| Fresh meat | Fresh meat deboned | Guinea fowl | Human consumption | Yes | Comext |
| Fresh meat | Fresh or frozen meat on the bone | Guinea fowl | Human consumption | Yes | Comext |
| Fresh meat | Fresh or frozen meat deboned | Guinea fowl | Human consumption | Yes | Comext |
| Fresh meat | Fresh or frozen meat deboned or dried and salted meat | Poultry (undefined) | Human consumption | Yes | Comext |
| Fresh meat | Fresh meat deboned | Poultry (undefined) | Human consumption | Yes | Comext |
| Fresh meat | Fresh or frozen meat deboned | Birds other than poultry | Human consumption | Yes | Comext |
| Frozen meat | Frozen meat deboned | Equines | Human consumption | Yes | Comext |
| Frozen meat | Frozen meat on the bone incl. carcasses | Bovines | Human consumption | Yes | Comext |

**Table S2.2.** Continued.

| **Summarizing product group** | **Product** | **Animal species** | **Destination** | **Slaughtered animal** | **Database** |
| --- | --- | --- | --- | --- | --- |
| Frozen meat | Frozen meat deboned | Bovines | Human consumption | Yes | Comext |
| Frozen meat | Frozen meat on the bone incl. carcasses | Pigs | Human consumption | Yes | Comext |
| Frozen meat | Frozen meat deboned | Pigs | Human consumption | Yes | Comext |
| Frozen meat | Frozen meat on the bone incl. carcasses | Sheep | Human consumption | Yes | Comext |
| Frozen meat | Frozen meat deboned | Sheep | Human consumption | Yes | Comext |
| Frozen meat | Frozen meat on the bone incl. carcasses | Goats | Human consumption | Yes | Comext |
| Frozen meat | Frozen meat deboned | Goats | Human consumption | Yes | Comext |
| Frozen meat | Frozen meat deboned | Sheep/goats | Human consumption | Yes | Comext |

**Table S2.2.** Continued.

| **Summarizing product group** | **Product** | **Animal species** | **Destination** | **Slaughtered animal** | **Database** |
| --- | --- | --- | --- | --- | --- |
| Frozen meat | Frozen meat deboned | Livestock mammals (undefined) | Human consumption | Yes | Comext |
| Frozen meat | Frozen meat on the bone incl. carcasses | Chicken | Human consumption | Yes | Comext |
| Frozen meat | Frozen meat deboned | Chicken | Human consumption | Yes | Comext |
| Frozen meat | Frozen meat on the bone incl. carcasses | Turkey | Human consumption | Yes | Comext |
| Frozen meat | Frozen meat deboned | Turkey | Human consumption | Yes | Comext |
| Frozen meat | Frozen meat on the bone incl. carcasses | Ducks | Human consumption | Yes | Comext |
| Frozen meat | Frozen meat deboned | Ducks | Human consumption | Yes | Comext |
| Frozen meat | Frozen meat on the bone incl. carcasses | Geese | Human consumption | Yes | Comext |

**Table S2.2.** Continued.

| **Summarizing product group** | **Product** | **Animal species** | **Destination** | **Slaughtered animal** | **Database** |
| --- | --- | --- | --- | --- | --- |
| Frozen meat | Frozen meat deboned | Geese | Human consumption | Yes | Comext |
| Dried and salted meat | Dried and salted meat | Equines | Human consumption | Yes | Comext |
| Dried and salted meat | Dried and salted meat | Bovines | Human consumption | Yes | Comext |
| Dried and salted meat | Dried and salted meat | Pigs | Human consumption | Yes | Comext |
| Dried and salted meat | Dried and salted meat | Sheep/goats | Human consumption | Yes | Comext |
| Dried and salted meat | Dried and salted meat | Artiodactyla | Human consumption | Yes | Comext |
| Dried and salted meat | Dried and salted meat | Livestock mammals (undefined) | Human consumption | Yes | Comext |
| Dried and salted meat | Dried and salted meat | Other mammals | Human consumption | Yes | Comext |

**Table S2.2.** Continued.

| **Summarizing product group** | **Product** | **Animal species** | **Destination** | **Slaughtered animal** | **Database** |
| --- | --- | --- | --- | --- | --- |
| Dried and salted meat | Dried and salted meat | Chicken | Human consumption | Yes | Comext |
| Dried and salted meat | Dried and salted meat | Ducks | Human consumption | Yes | Comext |
| Heated meat | Heated meat (min 70 degrees for 30 min) | Bovines | Human consumption | Yes | Comext |
| Heated meat | Heated meat (min 70 degrees for 30 min) | Pigs | Human consumption | Yes | Comext |
| Heated meat | Heated meat (min 70 degrees for 30 min) | Sheep | Human consumption | Yes | Comext |
| Heated meat | Heated meat (min 70 degrees for 30 min) | Goats | Human consumption | Yes | Comext |
| Heated meat | Heated meat (min 70 degrees for 30 min) | All livestock animals (mammals and poultry) | Human consumption | Yes | Comext |
| Heated meat | Pet food | Livestock mammals (undefined) | Pet food | Yes | Comext |

**Table S2.2.** Continued.

| **Summarizing product group** | **Product** | **Animal species** | **Destination** | **Slaughtered animal** | **Database** |
| --- | --- | --- | --- | --- | --- |
| Heated meat | Heated meat (min 70 degrees for 30 min) | Other mammals | Human consumption | Yes | Comext |
| Heated meat | Heated meat (min 70 degrees for 30 min) | Chicken | Human consumption | Yes | Comext |
| Heated meat | Heated meat (min 70 degrees for 30 min) | Poultry other than chicken | Human consumption | Yes | Comext |
| Milk/dairy products^a^ | Milk/dairy products | Bovines/sheep/goats | Human consumption | No | Comext |
| Milk/dairy products^b^ | Milk/dairy products | Bovines/sheep/goats | Animal feed | No | Comext |
| Table eggs/egg products | Eggs for human consumption | Chicken | Human consumption | No | Comext |
| Table eggs/egg products | Egg products of poultry | Chicken | Human consumption | No | Comext |
| Table eggs/egg products | Eggs for human consumption | Poultry other than chicken | Human consumption | No | Comext |
| Table eggs/egg products | Eggs for human consumption | Poultry (undefined) | Human consumption | No | Comext |

**Table S2.2.** Continued.

| **Summarizing product group** | **Product** | **Animal species** | **Destination** | **Slaughtered animal** | **Database** |
| --- | --- | --- | --- | --- | --- |
| Table eggs/egg products | Egg products of poultry | Poultry (undefined) | Human consumption | No | Comext |
| Table eggs/egg products | Eggs for human consumption | Birds other than poultry | Human consumption | No | Comext |
| Table eggs/egg products | Egg products of poultry | Birds other than poultry | Human consumption | No | Comext |
| Casings | Casings | Livestock mammals (undefined) | Human consumption | Yes | Comext |
| Hides | Raw hides and skins | Equines | Textiles | Yes | Comext |
| Hides | Semi-processed hides and skins (usual chemical and mechanical processes in tanning industry) | Equines | Textiles | Yes | Comext |
| Hides | Raw hides and skins | Equines/bovines | Textiles | Yes | Comext |
| Hides | Semi-processed hides and skins (usual chemical and mechanical processes in tanning industry) | Equines/bovines | Textiles | Yes | Comext |

**Table S2.2.** Continued.

| **Summarizing product group** | **Product** | **Animal species** | **Destination** | **Slaughtered animal** | **Database** |
| --- | --- | --- | --- | --- | --- |
| Hides | Wool (chemical treatment, or storage at specific temperature) | Equines/other mammals | Textiles | Yes | Comext |
| Hides | Raw hides and skins | Bovines | Textiles | Yes | Comext |
| Hides | Semi-processed hides and skins (usual chemical and mechanical processes in tanning industry) | Bovines | Textiles | Yes | Comext |
| Hides | Wool, hair (not treated) | Pigs | Textiles | Yes | Comext |
| Hides | Raw hides and skins | Pigs | Textiles | Yes | Comext |
| Hides | Semi-processed hides and skins (usual chemical and mechanical processes in tanning industry) | Pigs | Textiles | Yes | Comext |
| Hides | Raw hides and skins | Sheep | Textiles | Yes | Comext |
| Hides | Semi-processed hides and skins (usual chemical and mechanical processes in tanning industry) | Sheep | Textiles | Yes | Comext |
| Hides | Wool, hair (not treated) | Sheep | Textiles | No | Comext |

**Table S2.2.** Continued.

| **Summarizing product group** | **Product** | **Animal species** | **Destination** | **Slaughtered animal** | **Database** |
| --- | --- | --- | --- | --- | --- |
| Hides | Wool (chemical treatment, or storage at specific temperature) | Sheep | Textiles | No | Comext |
| Hides | Raw hides and skins | Goats | Textiles | Yes | Comext |
| Hides | Semi-processed hides and skins (usual chemical and mechanical processes in tanning industry) | Goats | Textiles | Yes | Comext |
| Hides | Wool, hair (not treated) | Camelidae/goats | Textiles | No | Comext |
| Hides | Wool, hair (not treated) | Sheep/goats/camelidae/other mammals | Textiles | No | Comext |
| Hides | Wool (chemical treatment, or storage at specific temperature) | Sheep/goats/camelidae/other mammals | Textiles | No | Comext |
| Hides | Wool, hair (not treated) | Other mammals | Textiles | Yes | Comext |
| Hides | Raw hides and skins | Other mammals | Textiles | Yes | Comext |
| Hides | Semi-processed hides and skins (usual chemical and mechanical processes in tanning industry) | Other mammals | Textiles | Yes | Comext |

**Table S2.2.** Continued.

| **Summarizing product group** | **Product** | **Animal species** | **Destination** | **Slaughtered animal** | **Database** |
| --- | --- | --- | --- | --- | --- |
| Hides | Raw hides and skins | Livestock mammals (undefined) | Textiles | Yes | Comext |
| Hides | Semi-processed hides and skins (usual chemical and mechanical processes in tanning industry) | Livestock mammals (undefined) | Textiles | Yes | Comext |
| Feathers and down | Feathers and down | Poultry (undefined) | Textiles | Yes | Comext |
| Feathers and down | Feathers and down | Birds other than poultry | Textiles | Yes | Comext |
| Bones | Fresh bones | Livestock mammals (undefined) | Other use | Yes | Comext |
| Bones | Dried or disinfected bones | Livestock mammals (undefined) | Other use | Yes | Comext |
| Bones | Fresh bones | Other mammals | Other use | Yes | Comext |
| Bones | Dried or disinfected bones | Other mammals | Other use | Yes | Comext |
| Bones | Bones | All live animals | Other use | No | Comext |
| Blood-meal, meat-and-bone meal, offal | Blood-meal, meat-and-bone meal, offal | All livestock animals (mammals and poultry) | On farm - crops and animals | Yes | Comext |

**Table S2.2.** Continued.

| **Summarizing product group** | **Product** | **Animal species** | **Destination** | **Slaughtered animal** | **Database** |
| --- | --- | --- | --- | --- | --- |
| Industrial use | Gelatine, collagen, tallow (incl. products for industrial use) | Bovines/sheep/goats | Industrial use | Yes | Comext |
| Industrial use | Gelatine, collagen, tallow (incl. products for industrial use) | Pigs | Industrial use | Yes | Comext |
| Industrial use | Gelatine, collagen, tallow (incl. products for industrial use) | Poultry (undefined) | Industrial use | Yes | Comext |
| Industrial use | Gelatine, collagen, tallow (incl. products for industrial use) | Livestock mammals (undefined) | Industrial use | Yes | Comext |
| Industrial use | Other industrial use | Livestock mammals (undefined) | Industrial use | Yes | Comext |
| Pharmaceutical use | Pharmaceutical use | All live animals | Pharmaceutical use | Yes | Comext |
| Litter and manure | Manure | Equines | On farm - crops | No | TRACES |
| Litter and manure | Manure | Bovines | On farm - crops | No | TRACES |
| Litter and manure | Manure | Pigs | On farm - crops | No | TRACES |
| Litter and manure | Manure | Sheep | On farm - crops | No | TRACES |

**Table S2.2.** Continued.

| **Summarizing product group** | **Product** | **Animal species** | **Destination** | **Slaughtered animal** | **Database** |
| --- | --- | --- | --- | --- | --- |
| Litter and manure | Manure | Goats | On farm - crops | No | TRACES |
| Litter and manure | Manure | Poultry (undefined) | On farm - crops | No | TRACES |
| Litter and manure | Manure | All live animals | On farm - crops | No | TRACES |

# ^a^ Milk and dairy products for human consumption.

^b^ Milk and dairy products as an ingredient of animal feed.

**Table S2.3.** Average weight per seizure $W_{CP}$ (kg) and relative proportion per product type ${RP}_{CP}$ (fraction) per source region for the pathways (animal product groups) considered for the traveler introduction route in RRAT. FFM=fresh and frozen meat; DSM=dried and salted meat; HM=heated meat. Sources: Defra, 2003-2010; VLA, 2004; Falk et al., 2013; Beutlich et al., 2015; Schoder et al., 2015.

|  | Caribbean | Central & South America | Central Africa | Eastern Africa | Eastern Asia | Eastern Europe | Near & Middle East | North America | Northern Africa | Oceania | South Eastern Asia | Southern Africa | Southern Asia | Western Africa |
| --- | --- | --- | --- | --- | --- | --- | --- | --- | --- | --- | --- | --- | --- | --- |
| Weight per seizure (kg) | 2.87 | 2.87 | 4.71 | 3.76 | 2.38 | 3.28 | 2.97 | 3.47 | 4.26 | 2.71 | 2.38 | 1.64 | 2.38 | 4.71 |
| Relative proportions per product type | | | | | | | | | | | | | | |
| FFM_bovine | 0.205 | 0.176 | 0.215 | 0.126 | 0.140 | 0.012 | 0.072 | 0.179 | 0.166 | 0.197 | 0.230 | 0.228 | 0.110 | 0.124 |
| DSM_bovine | 0.035 | 0.030 | 0.034 | 0.019 | 0.015 | 0.001 | 0.007 | 0.031 | 0.025 | 0.034 | 0.035 | 0.181 | 0.019 | 0.009 |
| HM_bovine | 0.053 | 0.045 | 0.020 | 0.011 | 0.023 | 0.000 | 0.013 | 0.045 | 0.014 | 0.036 | 0.039 | 0.013 | 0.019 | 0.016 |
| FFM_swine | 0.089 | 0.078 | 0.069 | 0.039 | 0.250 | 0.372 | 0.036 | 0.078 | 0.053 | 0.035 | 0.039 | 0.046 | 0.017 | 0.061 |
| DSM_swine | 0.013 | 0.013 | 0.011 | 0.006 | 0.027 | 0.032 | 0.003 | 0.013 | 0.008 | 0.004 | 0.005 | 0.036 | 0.002 | 0.005 |
| HM_swine | 0.023 | 0.020 | 0.006 | 0.004 | 0.042 | 0.006 | 0.006 | 0.020 | 0.004 | 0.006 | 0.003 | 0.002 | 0.003 | 0.008 |
| FFM_sheep | 0.006 | 0.005 | 0.057 | 0.033 | 0.004 | 0.004 | 0.062 | 0.005 | 0.044 | 0.065 | 0.077 | 0.008 | 0.034 | 0.017 |
| DSM_sheep | 0 | 0.001 | 0.009 | 0.005 | 4×10^-4^ | 3×10^-4^ | 0.006 | 0.001 | 0.007 | 0.001 | 0.010 | 0.006 | 0.006 | 0.001 |
| HM_sheep | 0.001 | 0.001 | 0.005 | 0.003 | 0.001 | 7×10^-5^ | 0.011 | 0.001 | 0.004 | 0.011 | 0.013 | 4×10^-4^ | 0.006 | 0.002 |
| FFM_goat | 0.005 | 0.005 | 0.058 | 0.033 | 0.008 | 0 | 0.021 | 0.005 | 0.044 | 0.012 | 0.014 | 0.008 | 0.007 | 0.024 |

**Table S2.3.** Continued.

|  | Caribbean | Central & South America | Central Africa | Eastern Africa | Eastern Asia | Eastern Europe | Near & Middle East | North America | Northern Africa | Oceania | South Eastern Asia | Southern Africa | Southern Asia | Western Africa |
| --- | --- | --- | --- | --- | --- | --- | --- | --- | --- | --- | --- | --- | --- | --- |
| DSM_goat | 0.000 | 0 | 0.009 | 0.005 | 0.001 | 0 | 0.002 | 0.000 | 0.006 | 0.002 | 0.002 | 0.006 | 0.001 | 0.002 |
| HM_goat | 0.001 | 0.001 | 0.005 | 0.003 | 0.001 | 0 | 0.004 | 0.001 | 0.004 | 0.002 | 0.002 | 0.000 | 0.001 | 0.003 |
| FFM_buffalo | 0.003 | 0.002 | 0.011 | 0.007 | 0.002 | 0 | 0 | 0.002 | 0.009 | 0.016 | 0.017 | 0 | 0.009 | 0.002 |
| DSM_buffalo | 4×10^-4^ | 0.000 | 0.002 | 0.001 | 2×10^-4^ | 0 | 0 | 4×10^-4^ | 0.001 | 0.002 | 0.002 | 0 | 0.001 | 0 |
| HM_buffalo | 0.001 | 0.001 | 0.001 | 0.001 | 1×10^-4^ | 0 | 0 | 0.001 | 0 | 0.003 | 0.002 | 0 | 0.001 | 0.000 |
| FFM_bushmeat | 0.005 | 0.004 | 0.069 | 0.040 | 0.002 | 0 | 0 | 0.005 | 0.053 | 0 | 0 | 0.027 | 0 | 0.261 |
| DSM_bushmeat | 0 | 0 | 0.011 | 0.006 | 2×10^-4^ | 0 | 0 | 0 | 0.008 | 0 | 0 | 0.021 | 0 | 0.020 |
| HM_bushmeat | 0.001 | 0.001 | 0.006 | 0.004 | 0 | 0 | 0 | 0.001 | 0.004 | 0 | 0 | 0.001 | 0 | 0.035 |
| Poultry meat | 0.288 | 0.250 | 0.330 | 0.192 | 0.340 | 0.286 | 0.161 | 0.253 | 0.252 | 0.270 | 0.313 | 0.356 | 0.150 | 0.181 |
| Milk & dairy products | 0.259 | 0.355 | 0.066 | 0.451 | 0.135 | 0.276 | 0.587 | 0.348 | 0.287 | 0.294 | 0.185 | 0.051 | 0.603 | 0.219 |
| Eggs | 0.010 | 0.010 | 0.010 | 0.010 | 0.010 | 0.010 | 0.010 | 0.010 | 0.010 | 0.010 | 0.010 | 0.010 | 0.010 | 0.010 |

# References

Beutlich J, Hammerl JA, Appel B, Nöckler K, Helmuth R, Jöst K, et al. Characterization of illegal food items and identification of foodborne pathogens brought into the European Union via two major German airports. *Internat J Food Microbiol*. (2015) 209:13-19. doi: 10.1016/j.ijfoodmicro.2014.10.017

CFSPH. The Center for Food Security and Animal Health, Iowa State University. (2022). Available online at: <https://www.cfsph.iastate.edu/diseaseinfo/> (accessed March 16, 2022).

Defra. Annual Review of Controls on Imports of Animal Products. April 2002 – March 2003. UK: Department for Environment Food and Rural Affairs. (2003). Available online at: <https://webarchive.nationalarchives.gov.uk/ukgwa/20060303120000/http:/www.defra.gov.uk/animalh/illegali/news.htm> (accessed December 16, 2019).

Defra. Annual Review of Controls on Imports of Animal Products. April 2003 – March 2004. UK: Department for Environment Food and Rural Affairs. (2004). Available online at: <https://webarchive.nationalarchives.gov.uk/ukgwa/20060303120000/http://www.defra.gov.uk/animalh/illegali/reports/index.htm> (accessed December 16, 2019).

Defra. Annual Review of Controls on Imports of Animal Products. April 2004 – March 2005. UK: Department for Environment Food and Rural Affairs. (2005). Available online at: <https://webarchive.nationalarchives.gov.uk/ukgwa/20060303120000/http://www.defra.gov.uk/animalh/illegali/reports/index.htm> (accessed December 16, 2019).

Defra. Annual Review of Controls on Imports of Animal Products. April 2005 – March 2006. UK: Department for Environment Food and Rural Affairs. (2006). Available online at: <https://webarchive.nationalarchives.gov.uk/20090903183114/http://www.official-documents.gov.uk/document/cm68/6897/6897.asp> (accessed December 16, 2019).

Defra. Annual Review of Controls on Imports of Animal Products. April 2006 – March 2007. UK: Department for Environment Food and Rural Affairs. (2007). Available online at: <https://webarchive.nationalarchives.gov.uk/20090903175233/http://www.official-documents.gov.uk/document/cm71/7179/7179.asp> (accessed December 16, 2019).

Defra. Annual Review of Controls on Imports of Animal Products. April 2007 – March 2008. UK: Department for Environment Food and Rural Affairs. (2008). Available online at: <https://webarchive.nationalarchives.gov.uk/20090903174059/http://www.official-documents.gov.uk/document/cm74/7444/7444.asp> (accessed December 16, 2019).

Defra. Annual Review of Controls on Imports of Animal Products. April 2008 – March 2009. UK: Department for Environment Food and Rural Affairs. (2009). Available online at: <https://webarchive.nationalarchives.gov.uk/ukgwa/20121205170130/http:/www.official-documents.gov.uk/document/other/9780101764827/9780101764827.asp> (accessed December 16, 2019).

Defra. Annual Review of Controls on Imports of Animal Products. April 2009 – March 2010. UK: Department for Environment Food and Rural Affairs. (2010). Available online at: <https://webarchive.nationalarchives.gov.uk/20121205161459/http://www.official-documents.gov.uk/document/other/9780102968576/9780102968576.asp> (accessed December 16, 2019).

Discontools. Research gaps for improving infectious disease control in animals. (2022). Available online at: <https://www.discontools.eu/database.html> (accessed March 16, 2022).

Dórea FC, Swanenburg M, Van Roermund H, Horigan V, De Vos C, Gale P, et al. Data collection for risk assessments on animal health. EFSA supporting publication (2017) EN-1171. doi: 10.2903/sp.efsa.2017.EN-1171.

EC (European Commission). TRACES: TRAde Control and Expert System. (2022). Available online at: <https://ec.europa.eu/food/animals/traces_en> (accessed March 16, 2022).

EFSA. EFSA Disease Profiles. (2022). Available online at: <https://animal-diseases.efsa.europa.eu/> (accessed March 16, 2022).

EU (European Union). Council Regulation (EEC) No 2658/87 of 23 July 1987 on the tariff and statistical nomenclature and on the Common Customs Tariff. *Off J Eur Union.* (1987) L256:1-1069. Consolidated version of 01/01/2021. ELI: <http://data.europa.eu/eli/reg/1987/2658/2021-01-01>

Eurostat. Comext Bulk Download. (2022). Available online at: <https://ec.europa.eu/eurostat/estat-navtree-portlet-prod/BulkDownloadListing?sort=1&dir=comext> (accessed March 16, 2022).

Falk H, Dürr S, Hauser R, Wood K, Tenger B, Lörtscher M, et al. Illegal import of bushmeat and other meat products into Switzerland on commercial passenger flights. *Rev Sci Tech.* (2013) 32:727-739.

OIE. Terrestrial Animal Health Code. World Organisation for Animal Health. (2021a). Available online at: <https://www.oie.int/en/what-we-do/standards/codes-and-manuals/terrestrial-code-online-access/> (accessed March 16, 2022).

OIE. Manual of Diagnostic Tests and Vaccines for Terrestrial Animals. World Organisation for Animal Health. (2021b). Available online at: <https://www.oie.int/en/what-we-do/standards/codes-and-manuals/terrestrial-manual-online-access/> (accessed March 16, 2022).

OIE. Animal Diseases. World Organisation for Animal Health. (2022). Available online at: <https://www.oie.int/en/what-we-do/animal-health-and-welfare/animal-diseases/> (accessed March 16, 2022).

Schoder D, Strauss A, Szakmary-Brändle K, Stessl B, Schlager S, Wagner, M. Prevalence of major foodborne pathogens in food confiscated from air passenger luggage. *Internat J Food Microbiol.* (2015) 209:3-12. doi: 10.1016/j.ijfoodmicro.2014.08.010

VLA. Risk assessment for the illegal import of meat and meat products contaminated with Foot and Mouth Disease (FMD), 2004. UK: Centre for Epidemiology and Risk Analysis, Veterinary Laboratories Agency. (2004). Available online at: <https://webarchive.nationalarchives.gov.uk/ukgwa/20060303120000/http:/www.defra.gov.uk/animalh/illegali/reports/index.htm> (accessed March 16, 2022).
