# Supplementary material for: Rapid risk assessment tool (RRAT) to prioritize emerging and re-emerging livestock diseases for risk management
Source: Front Vet Sci. 2022 Sep 7;9:963758. doi: 10.3389/fvets.2022.963758 (PMC9490411; doi:10.3389/fvets.2022.963758)
Supplement: Supplementary file 3 [file Table_3.docx]

Supplementary Material 3: Input parameters in RRAT

**Table S3.1.** Proxy values to account for differences in disease susceptibility between host types when considering the probability of infection (${P_{infsus}}_{P}$) and the probability of transmission ($P_{{trans}_{PD}}$). Values were based on expert opinion using a log_10_ scale for ${P_{infsus}}_{P}$ and a log_√10_ scale for $P_{{trans}_{PD}}$.

| **Host type** | ${\boldsymbol{P}_{\boldsymbol{infsus}}}_{\boldsymbol{P}}$ | $\boldsymbol{P}_{\boldsymbol{trans}_{\boldsymbol{PD}}}$ |
| --- | --- | --- |
| Reservoir host | 1 | 1 |
| Spill-over host | 0.01 | 0.3 |
| Experimental host | 0.001 | 0.1 |
| Dead-end host | 0.01 | 0 |
| Not susceptible | 0 | 0 |

**Table S3.2**. Disease-related proxy values for the infectious period indicated as fraction of the year (${T_{inf}}_{D}$), the minimum expected incidence (${{Inc}_{abs}}_{D}$), the maximum expected incidence (${{Inc}_{unk}}_{D}$), the period of absence of disease (in years) to have confidence in freedom of disease ($X_{D}$), and the estimated number of cases per outbreak based on OIE data (OIE, 2022a) ($C_{D}$).

| **Disease** | ${\boldsymbol{T}_{\boldsymbol{inf}}}_{\boldsymbol{D}}$ ^a^ | ${\boldsymbol{Inc}_{\boldsymbol{abs}}}_{\boldsymbol{D}}$^b^ | ${\boldsymbol{Inc}_{\boldsymbol{unk}}}_{\boldsymbol{D}}$^c^ | $\boldsymbol{X}_{\boldsymbol{D}}$ ^d^ | $\boldsymbol{C}_{\boldsymbol{D}}$ ^e^ |
| --- | --- | --- | --- | --- | --- |
| AHS | 0.05 | 10^-7^ | 10^-3^ | 3 | 1 |
| ASF | 0.1 | 10^-8^ | 10^-2^ | 3 | 30 |
| Auj | 0.05 | 10^-9^ | 10^-3^ | 10 | 3 |
| BT | 0.1 | 10^-9^ | 10^-2^ | 3 | 1 |
| bTB | 1 | 10^-10^ | 10^-3^ | 10 | 3 |
| CSF | 0.1 | 10^-9^ | 10^-2^ | 3 | 10 |
| EIA | 1 | 10^-8^ | 10^-2^ | 10 | 1 |
| FMD | 0.05 | 10^-9^ | 10^-2^ | 3 | 10 |
| LSD | 0.05 | 10^-10^ | 10^-2^ | 3 | 10 |
| PPR | 0.1 | 10^-9^ | 10^-3^ | 3 | 10 |

AHS=African horse sickness; ASF=African swine fever; Auj=Aujeszky’s disease; BT=bluetongue; bTB=bovine tuberculosis; CSF=classical swine fever; EIA=equine infectious anemia; FMD=foot-and-mouth disease; LSD=lumpy skin disease; PPR=peste des petits ruminants

^a^ $T_{{inf}_{D}}$ = 0.05 if infectious period < 2 weeks; $T_{{inf}_{D}}$ = 0.1 if infectious period > 2 weeks and < 1 month; $T_{{inf}_{D}}$ = 0.25 if infectious period > 1 month and < 1 year; $T_{{inf}_{D}}$ = 1 if infectious period > 1 year.

^b^ ${Inc}_{{abs}_{D}}$ is 100 times lower than the minimum incidence calculated for countries in risk class 4 (disease present and cases reported).

^c^ ${Inc}_{{unk}_{D}}$ is the maximum incidence calculated for countries in risk class 4.

^d^ $X_{D}$=10 if detection is likely to be hampered by a long incubation period of the disease and/or the presence of subclinically infected animals or carriers; $X_{D}$=3 otherwise.

^e^ $C_{D}$ is given on a log_√10_ scale; values were estimated based on the median number of cases per outbreak as reported by WAHIS and rounded to the nearest proxy value.

**Table S3.3.** Proxy values for the probability of contact of imported infected animal with susceptible livestock in the target area (${P_{contact}}_{P}$). Values were based on expert opinion using a log_10_ scale.

| **Destination** | **Animal type** | **Reservoir host** | **Other susceptibility classes** |
| --- | --- | --- | --- |
| Livestock farm | Livestock destined for life | 1 | 0.1 |
| Slaughterhouse | Livestock destined for slaughter | 0.01 | 0.01 |
| Household | Pets (dogs and cats) | 0.1 | 0.1 |
| Trade and approved bodies | Exotic mammals and birds | 0.1 | 0.1 |

**Table S3.4.** Proxy values for the probability tha0074 an infected animal is detected at slaughter by ante-mortem or post-mortem inspection ($P_{{detsl}_{D}}$). Values were based on expert opinion considering clinical signs and pathogenesis of disease and legal requirements.

| **Animal species group** | **Category** | **AHS** | **ASF** | **Auj** | **BT** | **bTB** | **CSF** | **EIA** | **FMD** | **LSD** | **PPR** |
| --- | --- | --- | --- | --- | --- | --- | --- | --- | --- | --- | --- |
| Live horses, asses, mules, hinnies destined for life | Equines | 0.3 | 0 | 0 | 0 | 0 | 0 | 0.1 | 0 | 0 | 0 |
| Live horses, asses, mules, hinnies destined for slaughter | Equines | 0.3 | 0 | 0 | 0 | 0 | 0 | 0.1 | 0 | 0 | 0 |
| Live bovines destined for life | Bovines | 0 | 0 | 0 | 0.1 | 0 | 0 | 0 | 0.5 | 0.7 | 0 |
| Live bovines destined for slaughter | Bovines | 0 | 0 | 0 | 0.1 | 0 | 0 | 0 | 0.5 | 0.7 | 0 |
| Live swine destined for life | Swine | 0 | 0.7 | 0 | 0 | 0 | 0.5 | 0 | 0.5 | 0 | 0 |
| Live swine destined for slaughter | Swine | 0 | 0.7 | 0 | 0 | 0 | 0.5 | 0 | 0.5 | 0 | 0 |
| Sheep destined for life | Small ruminants | 0 | 0 | 0 | 0.7 | 0 | 0 | 0 | 0.1 | 0 | 0.7 |
| Sheep destined for slaughter | Small ruminants | 0 | 0 | 0 | 0.7 | 0 | 0 | 0 | 0.1 | 0 | 0.7 |
| Goats destined for life | Small ruminants | 0 | 0 | 0 | 0.7 | 0 | 0 | 0 | 0.1 | 0 | 0.7 |
| Goats destined for slaughter | Small ruminants | 0 | 0 | 0 | 0.7 | 0 | 0 | 0 | 0.1 | 0 | 0.7 |
| Chickens (gallus domesticus) destined for life | Poultry | 0 | 0 | 0 | 0 | 0 | 0 | 0 | 0 | 0 | 0 |
| Chickens (gallus domesticus) destined for slaughter | Poultry | 0 | 0 | 0 | 0 | 0 | 0 | 0 | 0 | 0 | 0 |
| Turkeys destined for life | Poultry | 0 | 0 | 0 | 0 | 0 | 0 | 0 | 0 | 0 | 0 |
| Turkeys destined for slaughter | Poultry | 0 | 0 | 0 | 0 | 0 | 0 | 0 | 0 | 0 | 0 |
| Ducks destined for life | Poultry | 0 | 0 | 0 | 0 | 0 | 0 | 0 | 0 | 0 | 0 |

**Table S3.4.** Continued.

| **Animal species group** | **Category** | **AHS** | **ASF** | **Auj** | **BT** | **bTB** | **CSF** | **EIA** | **FMD** | **LSD** | **PPR** |
| --- | --- | --- | --- | --- | --- | --- | --- | --- | --- | --- | --- |
| Ducks destined for slaughter | Poultry | 0 | 0 | 0 | 0 | 0 | 0 | 0 | 0 | 0 | 0 |
| Geese destined for life | Poultry | 0 | 0 | 0 | 0 | 0 | 0 | 0 | 0 | 0 | 0 |
| Geese destined for slaughter | Poultry | 0 | 0 | 0 | 0 | 0 | 0 | 0 | 0 | 0 | 0 |
| Guinea fowls destined for life | Poultry | 0 | 0 | 0 | 0 | 0 | 0 | 0 | 0 | 0 | 0 |
| Guinea fowls destined for slaughter | Poultry | 0 | 0 | 0 | 0 | 0 | 0 | 0 | 0 | 0 | 0 |
| Primates | Other mammals | 0 | 0 | 0 | 0 | 0 | 0 | 0 | 0 | 0 | 0 |
| Sea mammals | Other mammals | 0 | 0 | 0 | 0 | 0 | 0 | 0 | 0 | 0 | 0 |
| Camelidae | Other mammals | 0 | 0 | 0 | 0.1 | 0 | 0 | 0 | 0.1 | 0 | 0.5 |
| Rabbits and hares | Other mammals | 0 | 0 | 0 | 0 | 0 | 0 | 0 | 0 | 0 | 0 |
| Dogs | Other mammals | 0 | 0 | 0 | 0 | 0 | 0 | 0 | 0 | 0 | 0 |
| Cats | Other mammals | 0 | 0 | 0 | 0 | 0 | 0 | 0 | 0 | 0 | 0 |
| Ferrets | Other mammals | 0 | 0 | 0 | 0 | 0 | 0 | 0 | 0 | 0 | 0 |
| Carnivora other than dogs, cats, ferrets | Other mammals | 0 | 0 | 0 | 0 | 0 | 0 | 0 | 0 | 0 | 0 |
| Artiodactyla | Other mammals | 0 | 0.1 | 0 | 0.1 | 0 | 0.3 | 0 | 0.1 | 0 | 0.5 |
| Perrisodactyla | Other mammals | 0.1 | 0 | 0 | 0 | 0 | 0 | 0 | 0 | 0 | 0 |
| Rodentia | Other mammals | 0 | 0 | 0 | 0 | 0 | 0 | 0 | 0 | 0 | 0 |

**Table S3.4.** Continued.

| **Animal species group** | **Category** | **AHS** | **ASF** | **Auj** | **BT** | **bTB** | **CSF** | **EIA** | **FMD** | **LSD** | **PPR** |
| --- | --- | --- | --- | --- | --- | --- | --- | --- | --- | --- | --- |
| Other mammals | Other mammals | 0 | 0 | 0 | 0 | 0 | 0 | 0 | 0 | 0 | 0 |
| Birds of prey | Other birds | 0 | 0 | 0 | 0 | 0 | 0 | 0 | 0 | 0 | 0 |
| Psittaciformes | Other birds | 0 | 0 | 0 | 0 | 0 | 0 | 0 | 0 | 0 | 0 |
| Ostriches and emus destined for life | Other birds | 0 | 0 | 0 | 0 | 0 | 0 | 0 | 0 | 0 | 0 |
| Ostriches and emus destined for slaughter | Other birds | 0 | 0 | 0 | 0 | 0 | 0 | 0 | 0 | 0 | 0 |
| Pigeons | Other birds | 0 | 0 | 0 | 0 | 0 | 0 | 0 | 0 | 0 | 0 |
| Other birds | Other birds | 0 | 0 | 0 | 0 | 0 | 0 | 0 | 0 | 0 | 0 |

AHS=African horse sickness; ASF=African swine fever; Auj=Aujeszky’s disease; BT=bluetongue; bTB=bovine tuberculosis; CSF=classical swine fever; EIA=equine infectious anemia; FMD=foot-and-mouth disease; LSD=lumpy skin disease; PPR=peste des petits ruminants

**Table S3.5.** Proxy values for the probability that an animal product is contaminated ($P_{{cont}_{PD}}$), given as an absence/presence score. Please note that pathogens can only be present in products derived from susceptible animal species. Scores were based on data derived from OIE (OIE, 2021a; OIE, 2021b; OIE, 2022b), factsheets (Dórea et al., 2017; CFSPH, 2022; Discontools, 2022; EFSA, 2022) and a review of scientific literature.

| **Summarizing product group** | **AHS** | **ASF** | **Auj** | **BT** | **bTB** | **CSF** | **EIA** | **FMD** | **LSD** | **PPR** |
| --- | --- | --- | --- | --- | --- | --- | --- | --- | --- | --- |
| Germplasm | 1 | 1 | 1 | 1 | 1 | 1 | 1 | 1 | 1 | 1 |
| Hatching eggs | 0 | 0 | 0 | 0 | 0 | 0 | 0 | 0 | 0 | 0 |
| Fresh meat | 1 | 1 | 1 | 1 | 1 | 1 | 0 | 1 | 0 | 1 |
| Frozen meat | 1 | 1 | 1 | 1 | 1 | 1 | 0 | 1 | 0 | 1 |
| Dried and salted meat | 0 | 1 | 0 | 1 | 0 | 1 | 0 | 1 | 0 | 1 |
| Heated meat | 0 | 0 | 0 | 0 | 0 | 0 | 0 | 0 | 0 | 0 |
| Milk and dairy products | 0 | 0 | 0 | 0 | 1 | 0 | 0 | 1 | 1 | 1 |
| Table eggs and egg products | 0 | 0 | 0 | 0 | 0 | 0 | 0 | 0 | 0 | 0 |
| Casings | 0 | 1 | 0 | 0 | 0 | 1 | 0 | 1 | 0 | 0 |
| Hides | 0 | 1 | 0 | 0 | 0 | 1 | 0 | 1 | 1 | 1 |
| Feathers and down | 0 | 0 | 0 | 0 | 0 | 0 | 0 | 0 | 0 | 0 |
| Bones | 0 | 1 | 1 | 1 | 0 | 1 | 0 | 1 | 0 | 0 |
| Blood-meal, MBM, offal | 0 | 0 | 0 | 0 | 0 | 0 | 0 | 0 | 0 | 0 |

**Table S3.5.** Continued.

| **Summarizing product group** | **AHS** | **ASF** | **Auj** | **BT** | **bTB** | **CSF** | **EIA** | **FMD** | **LSD** | **PPR** |
| --- | --- | --- | --- | --- | --- | --- | --- | --- | --- | --- |
| Industrial use | 0 | 0 | 0 | 0 | 0 | 0 | 0 | 0 | 0 | 0 |
| Pharmaceutical use | 1 | 1 | 0 | 1 | 0 | 1 | 1 | 1 | 1 | 1 |
| Litter and manure | 0 | 1 | 1 | 0 | 1 | 1 | 0 | 1 | 0 | 1 |

AHS=African horse sickness; ASF=African swine fever; Auj=Aujeszky’s disease; BT=bluetongue; bTB=bovine tuberculosis; CSF=classical swine fever; EIA=equine infectious anemia; FMD=foot-and-mouth disease; LSD=lumpy skin disease; PPR=peste des petits ruminants

**Table S3.6.** Proxy values for the probability of exposure (${P_{ex}}_{P}$) of local livestock to imported animal products and the most likely transmission route upon exposure based on intended use of the products. Values were based on expert opinion using a log_√10_ scale.

| **Intended use of product** | ${\boldsymbol{P}_{\boldsymbol{ex}}}_{\boldsymbol{P}}$ | **Exposure route** |
| --- | --- | --- |
| Breeding | 1 | Venereal/sexual |
| Human consumption | 0.01 | Oral ingestion |
| Pet food | 0.01 | Oral ingestion |
| Animal feed | 1 | Oral ingestion |
| On farm - livestock | 0.1 | Oral ingestion |
| On farm – crops (fertilizer) | 0.01 | Aerosols |
| On farm - crops and animals (MBM) | 0.03 | Oral ingestion |
| Textiles | 0.001 | Aerosols |
| Industrial use | 0.01 | Oral ingestion |
| Pharmaceutical use | 0.001 | Injection |
| Other use (bones, trophies) | 0.001 | Aerosols |

**Table S3.7.** Risk levels for the probability that animal products contain viable pathogen upon exposure to local animals ($P_{{contex}_{PD}}$) for the legal import of animal products. Risk levels were only assigned if $P_{{cont}_{PD}}=1$ (Table S3.5). Please note that pathogens can only be present in products derived from susceptible animal species. Risk levels were based on data derived from OIE (OIE, 2021a; OIE, 2021b; OIE, 2022b), factsheets (Dórea et al., 2017; CFSPH, 2022; Discontools, 2022; EFSA, 2022) and a review of scientific literature, using the criteria given in Fig. S3.1.

| **Summarizing product group** | **AHS** | **ASF** | **Auj** | **BT** | **bTB** | **CSF** | **EIA** | **FMD** | **LSD** | **PPR** |
| --- | --- | --- | --- | --- | --- | --- | --- | --- | --- | --- |
| Germplasm | Very low | Very low | Low | Low | Very low | Low | Very low | Moderate | Very low | Zero |
| Hatching eggs | 0 | 0 | 0 | 0 | 0 | 0 | 0 | 0 | 0 | 0 |
| Fresh meat | Very low | Moderate | Low | Very low | Low | Moderate | 0 | Moderate | 0 | Moderate |
| Frozen meat | Very low | Moderate | High | Very low | Low | Moderate | 0 | Moderate | 0 | Moderate |
| Dried and salted meat | 0 | Moderate | 0 | Very low | 0 | High | 0 | Moderate | 0 | Moderate |
| Heated meat | 0 | 0 | 0 | 0 | 0 | 0 | 0 | 0 | 0 | 0 |
| Milk and dairy products | 0 | 0 | 0 | 0 | Low | 0 | 0 | Moderate | Low | Low |
| Table eggs and egg products | 0 | 0 | 0 | 0 | 0 | 0 | 0 | 0 | 0 | 0 |
| Casings | 0 | Very low | 0 | 0 | 0 | Low | 0 | Low | 0 | 0 |
| Hides | 0 | Moderate | 0 | 0 | 0 | Moderate | 0 | High | Moderate | Very low |
| Feathers and down | 0 | 0 | 0 | 0 | 0 | 0 | 0 | 0 | 0 | 0 |
| Bones | 0 | Moderate | Moderate | Very low | 0 | Low | 0 | Moderate | 0 | 0 |

**Table S3.7.** Continued.

| **Summarizing product group** | **AHS** | **ASF** | **Auj** | **BT** | **bTB** | **CSF** | **EIA** | **FMD** | **LSD** | **PPR** |
| --- | --- | --- | --- | --- | --- | --- | --- | --- | --- | --- |
| Blood-meal, MBM, offal | 0 | 0 | 0 | 0 | 0 | 0 | 0 | 0 | 0 | 0 |
| Industrial use | 0 | 0 | 0 | 0 | 0 | 0 | 0 | 0 | 0 | 0 |
| Pharmaceutical use | Moderate | Moderate | 0 | High | High | Low | Moderate | High | Very low | Very low |
| Litter and manure | 0 | Moderate | Moderate | 0 | Moderate | Moderate | 0 | Moderate | 0 | Very low |

AHS=African horse sickness; ASF=African swine fever; Auj=Aujeszky’s disease; BT=bluetongue; bTB=bovine tuberculosis; CSF=classical swine fever; EIA=equine infectious anemia; FMD=foot-and-mouth disease; LSD=lumpy skin disease; PPR=peste des petits ruminants

**Table S3.8.** Probability scores for the risk classes used for the parameters probability of contamination at exposure (${P_{contex}}_{PD}$) and probability of infection upon exposure ($P_{{infex}_{PD}}$). Probability scores were based on a log_10_ scale.

| **Risk class** | **Probability score** |
| --- | --- |
| Very low | 0.001 |
| Low | 0.01 |
| Moderate | 0.1 |
| High | 1 |

**Table S3.9.** Risk of infection of a susceptible animal for each of the diseases per exposure route. Risk levels were based on data derived from OIE (OIE, 2021a; OIE, 2021b; OIE, 2022b), factsheets (Dórea et al., 2017; CFSPH, 2022; Discontools, 2022; EFSA, 2022) and a review of scientific literature.

| **Exposure route** | **AHS** | **ASF** | **Auj** | **BT** | **bTB** | **CSF** | **EIA** | **FMD** | **LSD** | **PPR** |
| --- | --- | --- | --- | --- | --- | --- | --- | --- | --- | --- |
| Venereal/sexual | Low | Low | Moderate | Low | Low | High | Low | Low | Low | Low |
| Oral ingestion | Low | High | High | Low | Moderate | High | Very low | Moderate | Low | Low |
| Aerosols | Low | Low | High | Very low | High | High | Very low | High | Moderate | High |
| Injection | High | High | Moderate | High | Moderate | High | High | High | High | High |

AHS=African horse sickness; ASF=African swine fever; Auj=Aujeszky’s disease; BT=bluetongue; bTB=bovine tuberculosis; CSF=classical swine fever; EIA=equine infectious anemia; FMD=foot-and-mouth disease; LSD=lumpy skin disease; PPR=peste des petits ruminants

**Table S3.10.** Risk levels for the probability that animal products contain viable pathogen upon exposure to local animals ($P_{{contex}_{PD}}$) for animal products carried by travelers. Risk levels were only assigned if $P_{{cont}_{PD}}=1$ (Table S3.5). Please note that pathogens can only be present in products derived from susceptible animal species. Risk levels were based on data derived from OIE (OIE, 2021a; OIE, 2021b; OIE, 2022b), factsheets (Dórea et al., 2017; CFSPH, 2022; Discontools, 2022; EFSA, 2022) and a review of scientific literature, using the criteria given in Fig. S3.1.

| **Product type** | **ASF** | **Auj** | **BT** | **bTB** | **CSF** | **FMD** | **LSD** | **PPR** |
| --- | --- | --- | --- | --- | --- | --- | --- | --- |
| FFM_bovine | 0 | 0 | Very low | Moderate | 0 | High | 0 | 0 |
| DSM_bovine | 0 | 0 | Very low | 0 | 0 | High | 0 | 0 |
| HM_bovine | 0 | 0 | 0 | 0 | 0 | 0 | 0 | 0 |
| FFM_swine | High | Low | 0 | Moderate | High | High | 0 | 0 |
| DSM_swine | High | 0 | 0 | 0 | High | High | 0 | 0 |
| HM_swine | 0 | 0 | 0 | 0 | 0 | 0 | 0 | 0 |
| FFM_sheep | 0 | 0 | Very low | 0 | 0 | High | 0 | High |
| DSM_sheep | 0 | 0 | Very low | 0 | 0 | High | 0 | High |
| HM_sheep | 0 | 0 | 0 | 0 | 0 | 0 | 0 | 0 |
| FFM_goat | 0 | 0 | Very low | Moderate | 0 | High | 0 | High |
| DSM_goat | 0 | 0 | Very low | 0 | 0 | High | 0 | High |
| HM_goat | 0 | 0 | 0 | 0 | 0 | 0 | 0 | 0 |
| FFM_buffalo | 0 | 0 | Very low | Moderate | 0 | High | 0 | 0 |
| DSM_buffalo | 0 | 0 | Very low | 0 | 0 | High | 0 | 0 |

**Table S3.10.** Continued.

| **Product type** | **ASF** | **Auj** | **BT** | **bTB** | **CSF** | **FMD** | **LSD** | **PPR** |
| --- | --- | --- | --- | --- | --- | --- | --- | --- |
| HM_buffalo | 0 | 0 | 0 | 0 | 0 | 0 | 0 | 0 |
| FFM_bushmeat | 0 | 0 | Very low | Moderate | 0 | High | 0 | 0 |
| DSM_bushmeat | 0 | 0 | Very low | 0 | 0 | High | 0 | 0 |
| HM_bushmeat | 0 | 0 | 0 | 0 | 0 | 0 | 0 | 0 |
| Poultry meat | 0 | 0 | 0 | 0 | 0 | 0 | 0 | 0 |
| Milk & dairy products | 0 | 0 | 0 | Moderate | 0 | High | Moderate | Moderate |
| Eggs | 0 | 0 | 0 | 0 | 0 | 0 | 0 | 0 |

**Fig. S3.1.** Criteria used to assess the probability that animal products are contaminated.


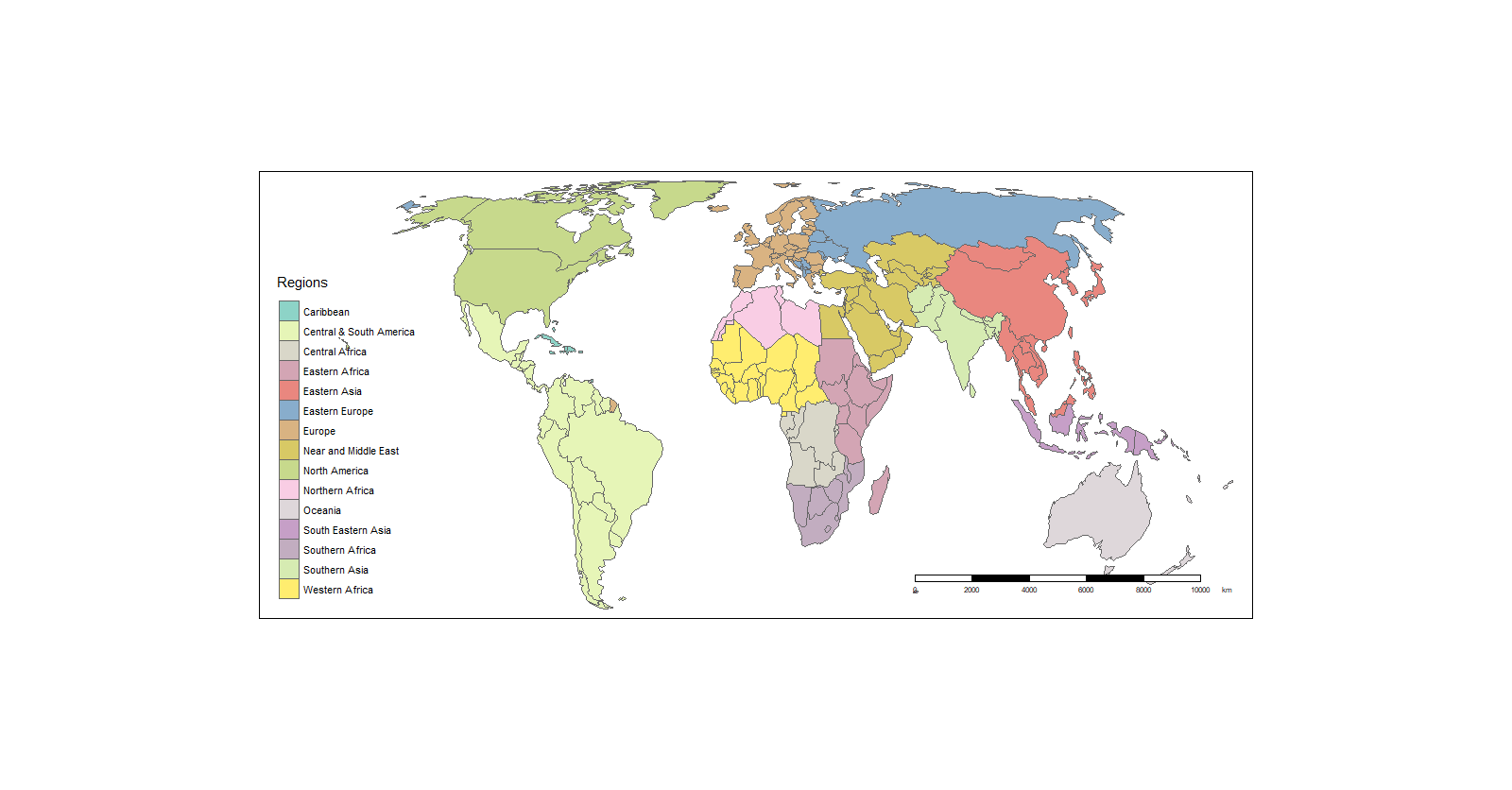


**Fig. S3.2.** Geographical regions used in the traveler pathway. For Europe (EU member states, EFTA countries and UK) no data was available on animal products carried by air travelers.

# References

CFSPH. The Center for Food Security and Animal Health, Iowa State University. (2022). Available online at: <https://www.cfsph.iastate.edu/diseaseinfo/> (accessed March 16, 2022).

Discontools. Research gaps for improving infectious disease control in animals. (2022). Available online at: <https://www.discontools.eu/database.html> (accessed March 16, 2022).

Dórea FC, Swanenburg M, Van Roermund H, Horigan V, De Vos C, Gale P, et al. Data collection for risk assessments on animal health. EFSA supporting publication (2017) EN-1171. doi: 10.2903/sp.efsa.2017.EN-1171.

EFSA. EFSA Disease Profiles. (2022). Available online at: <https://animal-diseases.efsa.europa.eu/> (accessed March 16, 2022).

OIE. Terrestrial Animal Health Code. World Organisation for Animal Health. (2021a). Available online at: <https://www.oie.int/en/what-we-do/standards/codes-and-manuals/terrestrial-code-online-access/> (accessed March 16, 2022).

OIE. Manual of Diagnostic Tests and Vaccines for Terrestrial Animals. World Organisation for Animal Health. (2021b). Available online at: <https://www.oie.int/en/what-we-do/standards/codes-and-manuals/terrestrial-manual-online-access/> (accessed March 16, 2022).

OIE. World Animal Health Information System. World Organisation for Animal Health. (2022a). Available online: <https://wahis.oie.int/#/home> (accessed March 16, 2022).

OIE. Animal Diseases. World Organisation for Animal Health. (2022b). Available online at: <https://www.oie.int/en/what-we-do/animal-health-and-welfare/animal-diseases/> (accessed March 16, 2022).
