# Supplementary material for: Rapid risk assessment tool (RRAT) to prioritize emerging and re-emerging livestock diseases for risk management
Source: Front Vet Sci. 2022 Sep 7;9:963758. doi: 10.3389/fvets.2022.963758 (PMC9490411; doi:10.3389/fvets.2022.963758)
Supplement: Supplementary file 4 [file Table_4.docx]

Supplementary Material 4: Sensitivity analysis

**Table S4.1**. Disease-related proxy values for the minimum expected prevalence (${P_{abs}}_{D}$), the maximum expected prevalence (${P_{unk}}_{D}$), and underreporting (${UF}_{D}$) as used in alternative scenarios 1B, 1C, and 1E, respectively.

| **Disease** | ${\boldsymbol{P}_{\boldsymbol{abs}}}_{\boldsymbol{D}}$ | ${\boldsymbol{P}_{\boldsymbol{unk}}}_{\boldsymbol{D}}$ | $\boldsymbol{UF}_{\boldsymbol{D}}$ |
| --- | --- | --- | --- |
| AHS | 10^-5^ | 0.1 | 2.5 |
| ASF | 10^-6^ | 0.1 | 2.5 |
| Auj | 10^-7^ | 0.3 | 4 |
| BT | 10^-7^ | 0.1 | 2.5 |
| bTB | 10^-8^ | 0.3 | 4 |
| CSF | 10^-7^ | 0.1 | 2.5 |
| EIA | 10^-6^ | 0.3 | 4 |
| FMD | 10^-7^ | 0.1 | 2.5 |
| LSD | 10^-8^ | 0.1 | 2.5 |
| PPR | 10^-7^ | 0.1 | 2.5 |

AHS=African horse sickness; ASF=African swine fever; Auj=Aujeszky’s disease; BT=bluetongue; bTB=bovine tuberculosis; CSF=classical swine fever; EIA=equine infectious anemia; FMD=foot-and-mouth disease; LSD=lumpy skin disease; PPR=peste des petits ruminants


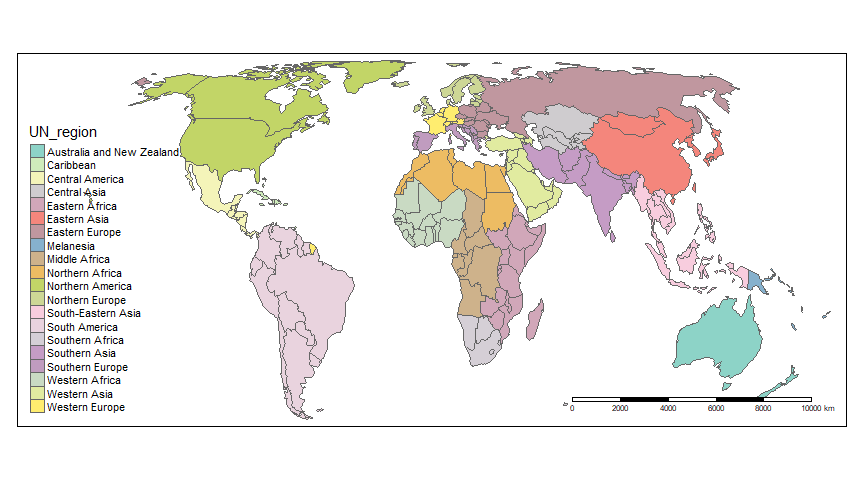


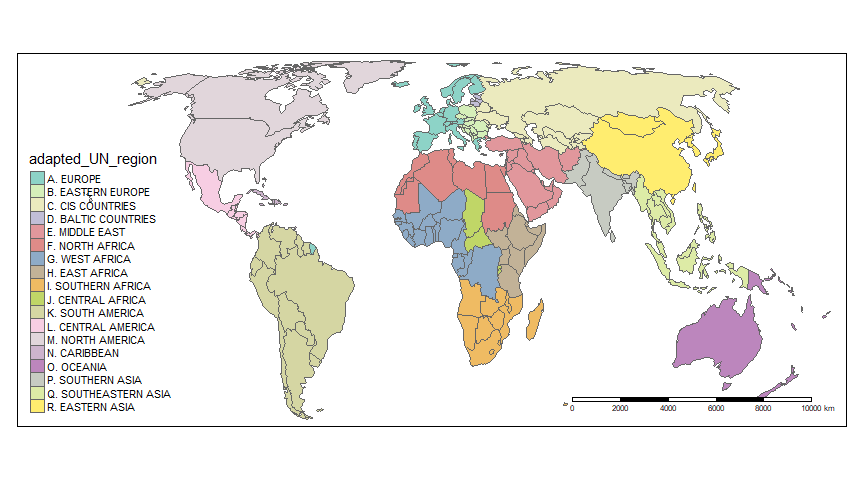


**B**

**Fig. S4.1.** A: UN subregions (UN, 2022) and B: Adjusted UN subregions (Simons et al., 2019).

**Fig. S4.2.** Spearman's rank correlation coefficients indicating the agreement in ranking of risk scores for individual pathways (x-axis, “category”) and individual diseases (y-axis, “disease”) between the baseline scenario and each alternative scenario.

**Fig. S4.3.** Spearman's rank correlation coefficients indicating the agreement in ranking of risk scores for pathways (x-axis, “category”), source countries^a^ (x-axis, “geo”) and diseases (y-axis, “diseases”) between years; 2016 was taken as the reference year.

# References

Simons RRL, Horigan V, Ip S, Taylor RA, Crescio MI, Maurella C, et al. A spatial risk assessment model framework for incursion of exotic animal disease into the European Union Member States. *Microbial Risk Anal.* (2019) 13:100075. doi: 10.1016/j.mran.2019.05.001

UN. UN Statistics Division. Standard country or area codes for statistical use (M49). United Nations. (2022). Available online at: <https://unstats.un.org/unsd/methodology/m49> (accessed March 15, 2022).
