# Supplementary material for: Rapid risk assessment tool (RRAT) to prioritize emerging and re-emerging livestock diseases for risk management
Source: Front Vet Sci. 2022 Sep 7;9:963758. doi: 10.3389/fvets.2022.963758 (PMC9490411; doi:10.3389/fvets.2022.963758)
Supplement: Supplementary file 5 [file Table_5.docx]

Supplementary Material 5: Qualitative risk levels

**Table S5.1.** Translation of probability-based risk scores into qualitative risk levels for the animal route.

| **Risk score** | **Qualitative risk level** | **Interpretation** |
| --- | --- | --- |
| ≥0.5 and ≤1 | Very high | An incursion will almost certainly occur |
| ≥ 0.1 and <0.5 | High | An incursion is likely to occur |
| ≥ 0.01 and <0.1 | Moderate | An incursion is possible, but not very likely |
| ≥0.001 and <0.01 | Low | An incursion is rare, but cannot be excluded |
| < 0.001 | Very low | An incursion is very rare, and can in practical terms be ignored |

**Table S5.2.** Translation of probability-based risk scores into qualitative risk levels for the product and traveler route.

| **Risk score** | **Qualitative risk level** | **Interpretation** |
| --- | --- | --- |
| ≥0.5 and ≤1 | High | An incursion is likely to occur |
| ≥ 0.1 and <0.5 | Moderate | An incursion is possible, but not very likely |
| ≥ 0.01 and <0.1 | Low | An incursion is rare, but cannot be excluded |
| <0.01 | Very low | An incursion is very rare, and can in practical terms be ignored |
